# Mechanistically Interpretable Artificial Intelligence for Designing Oxygen Electrocatalysts

**DOI:** 10.1002/adma.74212

**Published:** 2026-07-21

**Authors:** Xueyu Hu, Yucun Zhou, Haoyu Li, Zheyu Luo, Nai Shi, Yong Ding, Weining Wang, Weilin Zhang, Doyeub Kim, Chanho Kim, Yoojin Ahn, Nikhil Govindarajan, Minda Zou, Zhijun Liu, Yuefeng Song, Meilin Liu

**Affiliations:** ^1^ School of Materials Science and Engineering Georgia Institute of Technology Atlanta USA; ^2^ Kyushu University Platform of Inter‐/Transdisciplinary Energy Research Kyushu University Fukuoka Japan

**Keywords:** active learning, density functional theory, machine learning, oxygen electrocatalyst, solid oxide cell

## Abstract

Oxygen reduction and evolution reactions (ORR and OER) are key electrochemical processes central to energy conversion and chemical transformation. However, the inherently complex, multi‐physics nature of ORR/OER—together with diverse operating environments—poses significant challenges to the rational design of electrocatalysts based on structure–property relationships. To overcome these challenges, we developed Two‐Stage Material Screening (TSMS), an AI‐driven framework that integrates density functional theory (DFT) computations, an active‐learning‐guided experimental feedback loop, and mechanistic interpretation to enable rapid discovery and systematic evaluation of promising electrocatalysts. Demonstrated in protonic solid oxide cells (P‐SOCs), TSMS screened 6,940,032 compositions and identified top‐performing candidates that were experimentally validated, achieving a peak power density of 2.68 W cm^−2^ in fuel cell mode and a current density of 3.51 A cm^−2^ at 1.3 V in electrolysis mode, with stable performance maintained over 500 h at 600°C. Our analysis revealed that electron affinity is strongly associated with thermodynamic stability, while *d‐p* hybridization and densification resistance emerge as the primary descriptors governing electrocatalytic activity. By combining predictive modeling with mechanistic understanding, TSMS establishes a versatile and broadly generalizable paradigm for accelerating materials discovery.

## Graphical abstract

Mechanistically interpretable artificial intelligence screens nearly seven million perovskite compositions and identifies key descriptors—*d‐p* hybridization and densification resistance—that govern oxygen electrocatalysis. The discovered BaCo_0.8_Nb_0.1_Zr_0.1_O_3‐δ_ achieves a record 2.68 W cm^−2^ peak power density at 600°C with over 500 h of durable operation in protonic solid oxide cells.

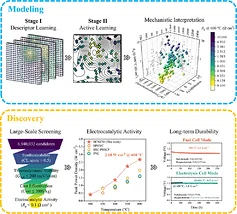

The escalating global energy crisis necessitates the rapid deployment of clean energy conversion and storage technologies, which are essential for curbing fossil‐fuel dependence, enabling efficient energy transformation, and ultimately achieving a zero‐emission future [1, 2, 3, 4]. Central to their transformation is the discovery of advanced ORR/OER electrocatalysts, which are foundational to the performance of fuel cells, metal‐air batteries, and reversible electrochemical systems [5, 6, 7, 8]. Protonic solid oxide cells (P‐SOCs) represent one of the most efficient platforms for power generation and green‐hydrogen production. However, progress remains constrained by the bottleneck of ORR/OER electrolysis, limited by slow experimental throughput, fragmented exploration of the vast chemical space, and the absence of unifying design principles [9]. To overcome this limitation and address the persistent activity‐stability trade‐off, it is essential to construct multi‐level descriptors—spanning defect chemistry, electronic structure, and thermodynamic properties—to uncover latent couplings and extract mechanistic insights into the underlying structure–property relationships [10].

Modern materials informatics has introduced a broad array of descriptors to guide the development of ORR/OER electrocatalysts [11, 12, 13, 14], including the energy above the convex hull (*E*_hull_) for thermodynamic stability, the oxygen *p*‐band center for charge transfer characteristics, and *e*_g_ orbital filling for adsorption kinetics [15, 16, 17]. Building on these efforts, AI‐driven surrogate models for first‐principles calculations—such as CHGNet, MACE, and NequIP—have substantially accelerated the evaluation of individual material properties, enabling rapid estimation of energetics and interatomic interactions with near‐DFT accuracy [9, 18, 19, 20, 21, 22]. More broadly, the emergence of open‐data platforms such as the Materials Project [23] and the Open Catalysts Project [24] has provided large‐scale, high‐fidelity computational datasets that underpin data‐driven research across diverse material systems. In parallel, the field has evolved from forward high‐throughput screening toward inverse design strategies enabled by deep generative models such as MatterGen [25], reinforcement learning, and active learning approaches [26]. In addition, domain‐knowledge‐informed active learning has shown particular promise for targeted materials discovery, where physically motivated constraints substantially reduce the experimental search space [27].

Despite these advances, the full potential of AI for ORR/OER electrocatalyst design remains constrained by the limited availability of high‐quality data and the high cost of generating catalysis‐relevant, high‐fidelity labels. To date, flagship repositories contain only 1,518 computed crystal structures [23] and 745 experimentally synthesized compounds [28], leaving vast regions of chemical space unexplored and most descriptors unlabeled. As a result, current AI applications in this field are largely restricted to surrogate modeling of individual properties, synthesizability evaluation, and prediction of fundamental properties—falling short of the integrated, multi‐objective frameworks required for complex electrocatalyst design. Given that ORR/OER electrocatalysts operate under complex, multidimensional physicochemical environments, their development demands an integrated and scalable workflow—one that unifies computation, experimentation, and artificial intelligence to expand the label space, enable accurate predictions, and uncover the governing physical principles.

To address these challenges, we developed an automated, closed‐loop Two‐Stage Material Screening (TSMS) framework (Figure 1) that enables rapid, large‐scale, and interpretable exploration of ORR/OER electrocatalysts with high predictive accuracy. The top‐performing candidates identified by TSMS significantly accelerated the computational down‐selection of oxygen electrodes for P‐SOCs, reducing the number of candidates requiring experimental synthesis and evaluation from hundreds to tens, and thereby substantially shortening the overall discovery timeline. Specifically, Stage Ⅰ addresses the challenge of sparse chemical coverage in descriptor spaces by integrating high‐throughput density functional theory (DFT) computations with transferable supervised learning. This approach enables the prediction of six key descriptors relevant to the ORR/OER across previously uncharted regions of chemical space. A multi‐objective optimization (MOOP) method is then applied to identify and recommend the most promising regions for initial experimental validation [29]. Stage Ⅱ reduces the experimental burden by implementing a Bayesian optimization strategy that maximizes expected information gain, thereby guiding standardized experiments toward the most informative candidates. Upon reaching a sampling space of 30,330 computational entries and 568 high‐fidelity experimental data points, the model enabled direct prediction of both thermodynamic stability and electrocatalytic activity, facilitating the identification of top‐performing candidates.

**FIGURE 1:**
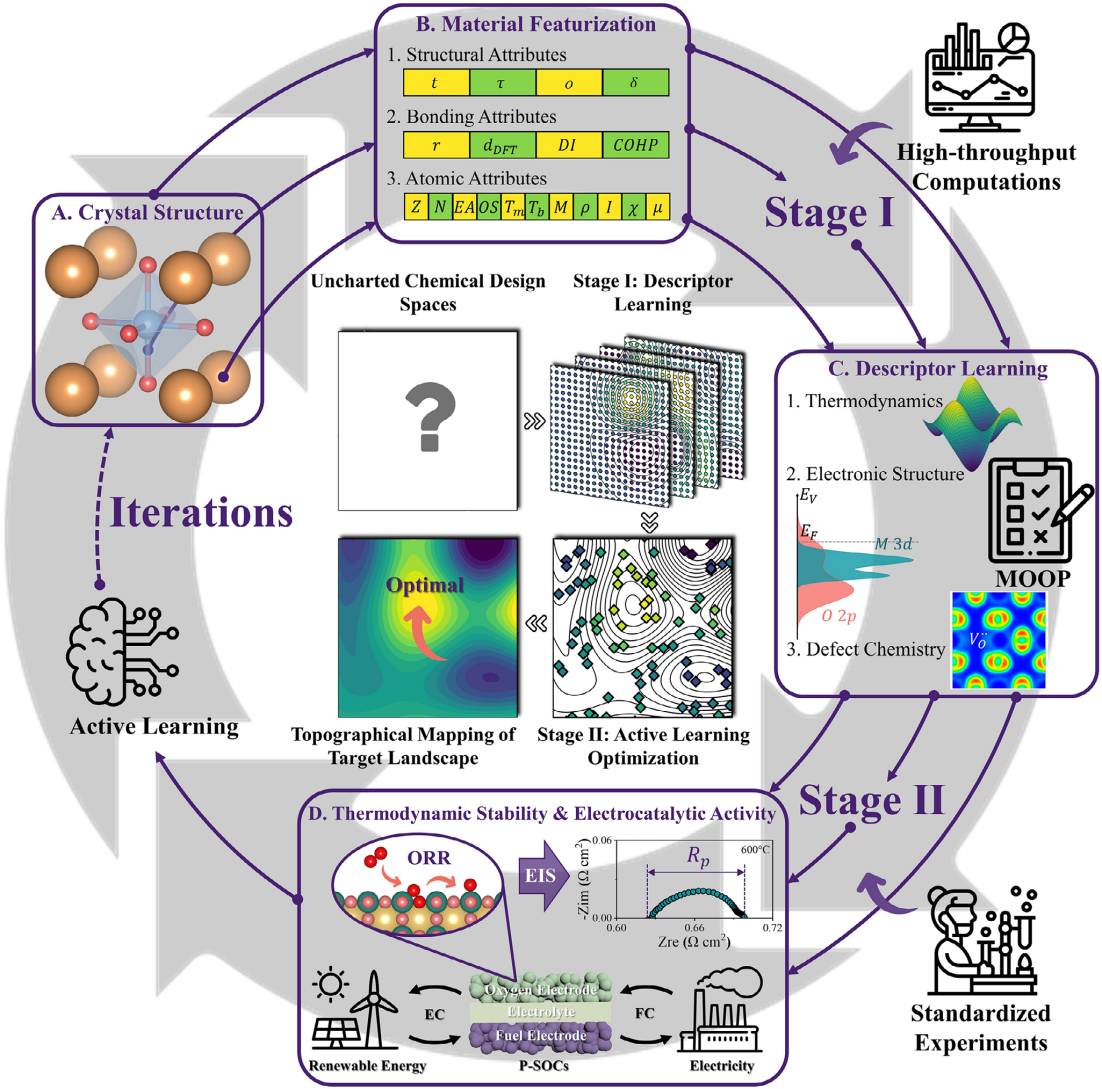
Two‐stage material screening (TSMS) framework. TSMS establishes an automated, closed‐loop workflow that overcomes the inefficiencies of traditional trial‐and‐error experimentation, dramatically accelerating both the speed and precision of large‐scale material screening. Targeting crystal structures in uncharted chemical design spaces, the framework begins with material featurization, extracting atomic, bonding, and structural attributes as input features. In Stage Ⅰ, a machine learning regression model trained on high‐throughput computations enables transferable descriptor prediction and global exploration of the design space. A multi‐objective optimization (MOOP) strategy identifies top‐ranked candidates, which form the initial seed set for Stage Ⅱ. Standardized experiments are then performed to evaluate their thermodynamic stability and electrocatalytic activity. Guided by an active learning model, Stage Ⅱ iteratively conducts closed‐loop experimental optimization by maximizing expected information gain. Through successive refinement, TSMS produces a high‐resolution topographical map of the target landscape and pinpoints truly optimal regions.

Mechanistically, TSMS constructs a high‐resolution topographic map of thermodynamic stability and electrocatalytic activity, quantified by the Gibbs free energy above the convex hull (*G*_hull_) and polarization resistance (*R*_p_), respectively. This approach achieves an order‐of‐magnitude improvement in predictive accuracy over state‐of‐the‐art methods [30, 31, 32]. Feature attribution analysis reveals that *G*_hull_ is most strongly associated with electron affinity, whereas *R*_p_ is primarily influenced by *d*–*p* hybridization (*H_dp_*) and densification resistance. These insights transform black‐box predictions into actionable design principles, offering new physical understanding of electrocatalyst performance. Through the systematic screening of 6,940,032 candidates, TSMS identified 4,287 promising compositions, over 20 of which were experimentally validated. Among these, five top‐performing materials enabled P‐SOCs to achieve a record‐breaking peak power density of 2.68 W cm^−2^—35% higher than current benchmarks—and an exceptional current density of 3.51 A cm^−2^ at 1.3 V and 600°C, with stable electrolysis performance sustained for over 500 h, validating the framework's predictive accuracy and technological advancement. More broadly, TSMS establishes a mechanistically interpretable, AI‐driven discovery paradigm that links intrinsic material properties with complex, multi‐physics‐coupled behaviors, paving the way for guided discovery across a broader landscape of energy materials.

## 1 Stage Ⅰ: Descriptor Learning

Stage Ⅰ established machine‐learning descriptors that capture physicochemical parameters governing both thermodynamic stability and ORR/OER electrocatalytic activity. Starting with state‐of‐the‐art perovskites, we systematically substituted A‐ and B‐site cations according to strict criteria for oxidation state, ionic radius compatibility, and charge neutrality. This expansion produced 6,066 unique compositions, 97% of which are absent from existing databases, thereby forming a broad chemical search space anchored to high‐performance prototypes (Supporting Note S1). We then performed high‐throughput DFT calculations to obtain ground‐truth ORR/OER descriptors for the entire set. These data were used to train a first‐stage machine learning (FSML) model that predicts descriptors orders of magnitude faster than DFT. FSML operates on a 168‐dimensional feature set that encodes atomic, bonding, and structural attributes grounded in perovskite crystal chemistry (Figure 1*A*: *Material Featurization*; Supporting Note S2 and Tables S1–S3).

**FIGURE 2:**
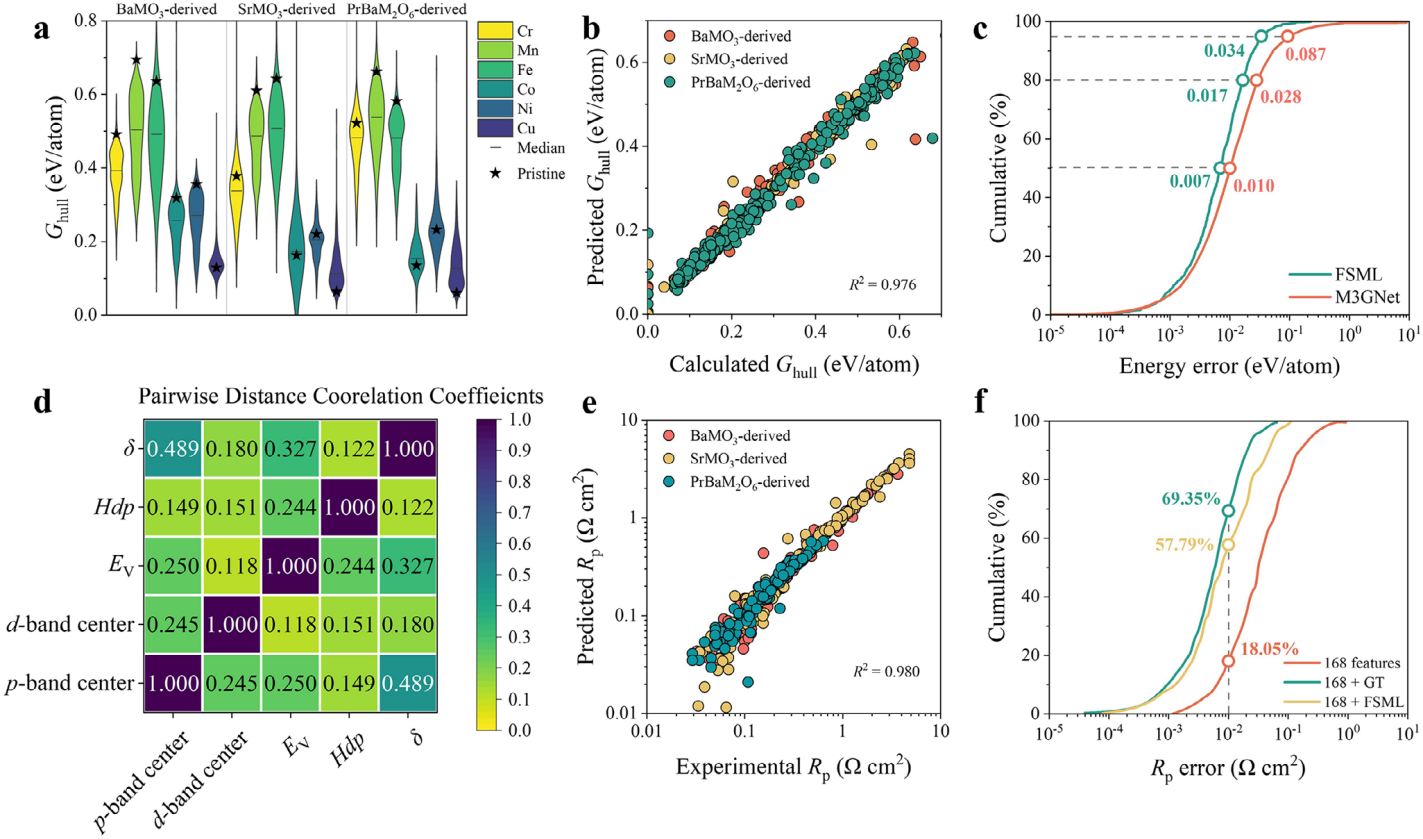
Performance of the machine learning models. (a) Violin plots showing the distribution of *G*_hull_ values across 18 compositional branches of perovskite candidates, with median and undoped pristine values annotated. (b) Parity plots comparing FSML‐predicted values versus DFT‐computed *G*_hull_ values. (c) Cumulative error distribution of *G*_hull_ predictions from FSML versus M3GNet models. (d) Pairwise distance correlation coefficients among the five activity descriptors, illustrating their orthogonality. (e) Parity plots of *R*_p_ predictions generated by the SSAL model. (f) Cumulative error distribution of *R*_p_ predictions by SSAL: "168 features" denotes the baseline model using only 168 physicochemical attributes; "168 + GT" includes both the 168 physicochemical features and ground‐truth descriptors obtained from DFT calculations; "168 + FSML" incorporates the 168 physicochemical features along with the FSML‐predicted descriptors.

FSML predicts thermodynamic stability with near‐DFT fidelity by directly modeling *G*_hull_ at T = 600°C and *p*O_2_ = 0.21 atm. Among 6066 candidates, grouped into 18 A‐/B‐site branches, *G*_hull_ exhibits a clear B‐site trend (Cu > Co > Ni > Cr > Fe ≈ Mn; Figure 2a). Benchmarking multiple learning algorithms on the physics‐informed, 168‐dimensional feature set, the optimized FSML model achieved an *R* ^2^ of 0.976 against DFT‐computed *G*_hull_ ground truth (Figure 2b), with 50%, 80%, and 95% of predictions within 0.007, 0.017, and 0.034 eV/atom, respectively (Figure 2c). This performance surpasses the state‐of‐the‐art M3GNet model [20], highlighting the value of comprehensive material featurization beyond conventional atomic‐triplet representations [25].

Concurrently, the ORR/OER electrocatalytic activity was elucidated through a suite of physically informed descriptors—including the oxygen *p*‐band center, transition‐metal *d*‐band center, oxygen vacancy formation energy (*E*_V_), oxygen non‐stoichiometry (δ), and *H_dp_*—with detailed definitions provided in Table S4 and Supporting Note S3. These descriptors capture key mechanistic aspects of the ORR/OER process from distinct physicochemical perspectives, encompassing electronic structure, defect chemistry, and mass transport [11, 17, 30, 33]. Their distributions across 18 compositional branches reveal distinct branch‐level signatures (Figure S1), underscoring chemically differentiated design spaces. Consistent with the performance observed for *G*_hull_, the FSML model demonstrated superior predictive accuracy across all descriptors when benchmarked against state‐of‐the‐art methods (Figure S2; Tables S5 and S6). Notably, for the computationally intensive descriptor *E*_V_, and even experimentally measured δ, FSML achieved DFT‐ and experiment‐level accuracy, respectively, with over 1000× acceleration.

To assess whether FSML generalizes across dopant chemistries rather than memorizing route‐specific patterns, we performed leave‐one‐route‐out (LORO) cross‐validation, in which all compositions from a given dopant route (i.e., the same host lattice and dopant pair across all doping fractions) are entirely excluded from training. Across all 337 routes, LORO achieved an *R* ^2^ of 0.920 and an RMSE of 0.025 eV/atom for *G*_hull_ (Table S5 and Supporting Note S2), confirming that the model captures transferable physicochemical trends.

To further evaluate the generalizability of our FSML model beyond the training domain, we constructed an out‐of‐distribution test set comprising 2022 LaMO_3_‐derived compositions. Uniform manifold approximation and projection (UMAP) analysis (Figure S3) revealed a clear distributional divergence between the training and target domains, highlighting the challenge of extrapolation. Remarkably, with fine‐tuning using only 1% of labeled samples from the target domain, FSML maintained high predictive accuracy (*R* ^2^ > 0.9) for *G*_hull_ and all ORR/OER electrocatalytic activity descriptors, demonstrating robust cross‐domain performance (Table S5 and Supporting Note S2). These results confirm that FSML captures transferable, physically meaningful descriptors for perovskite‐type materials. Such transferability stems from the combination of our high‐fidelity computational dataset and comprehensive featurization strategy, which form the foundation for the closed‐loop discovery and large‐scale screening efforts described in subsequent sections.

The five activity descriptors are largely orthogonal, as indicated by low pairwise distance correlation coefficients (mostly <0.5; Figure 2d), consistent with their distinct physical roles in governing ORR/OER performance. Such orthogonality reflects the multiscale interplay among surface exchange kinetics, bulk defect transport, interfacial charge transfer, and microstructural effects that collectively define the complexity of ORR/OER [34, 35, 36]. To directly predict the primary ORR/OER electrocatalytic activity indicator, *R*_p_ (typically determined from electrochemical impedance spectroscopy (EIS) of a well‐designed cell), it is essential to embed this descriptor diversity into feature design. These descriptors thus serve both as interpretable proxies for *R*_p_ and as transferable, mechanism‐aware representations for downstream modeling. Together, they establish the foundation for the integrative framework introduced in Stage Ⅱ, which aims to predict *R*_p_ directly by synthesizing these complementary physical insights.

## 2 Stage Ⅱ: Active Learning‐Accelerated Optimization

Stage Ⅱ enabled data‐efficient prediction of *R*_p_, a key experimental metric quantifying ORR/OER electrocatalytic activity, through an iterative, closed‐loop learning framework termed second‐stage active learning (SSAL; Supporting Note S4). The initial SSAL training dataset was derived from the top‐performing 1% of compositions, approximately 60 materials (240 entries), identified in Stage Ⅰ using a MOOP algorithm [29]. This algorithm ranks chemically meaningful candidates across the high‐dimensional descriptor space based on weighted, normalized descriptor vectors. The SSAL model was subsequently trained using the same 168‐dimensional feature set developed in Stage Ⅰ, augmented with descriptors predicted from the FSML model. As new experimental data were acquired, they were incorporated into the training set, the model was retrained, and updated predictions guided the selection of subsequent candidates.

Candidate selection in SSAL was guided by a Bayesian optimization strategy built on a Gaussian‐process surrogate and an expected‐improvement (EI) acquisition function [37]. The EI criterion jointly accounts for predicted *R*_p_ values and model uncertainty, balancing exploitation of promising low‐*R*_p_ candidates with exploration of under‐sampled regions of the composition space. In each acquisition round, candidates ranked highest by the EI score were selected for experimental synthesis and testing, and the resulting measurements were added to the training set to update the model. After seven acquisition rounds, the dataset expanded to 568 entries. Beyond this point, two quantitative convergence criteria were simultaneously satisfied: (1) the model training RMSE and *R* ^2^ for log(*R*_p_) plateaued; (2) the Spearman rank correlation between consecutive candidate rankings exceeded 0.95, confirming stabilization of the predicted performance landscape (Supporting Note S4, Figure S4, and Tables S7 and S8).

Figure 2e compares SSAL‐predicted and experimentally measured *R*_p_ values, yielding an *R* ^2^ of 0.980 and an RMSE of just 0.020 for log (*R*_p_) predictions—nearly an order of magnitude improvement over previously reported methods (Table S9). The cumulative error distribution (Figure 2f) shows that 57.79% of predictions fall within an absolute error of 0.01 Ω cm^2^, further demonstrating the model's precision. An ablation study assessed the value of FSML‐derived descriptors. Replacing FSML‐predicted descriptors with ground truth values improved the RMSE only marginally (from 0.020 to 0.015), indicating that FSML already provides high‐fidelity surrogates. In contrast, removing the descriptor set led to a sharp performance drop (RMSE = 0.110), confirming its critical role as a hyper‐feature scaffold that enhances model expressiveness and generalization. For *R*_p_ activation energy (*E*_a_), SSAL achieved *R* ^2^ of 0.996 and an RMSE of 0.028 eV (Figure S5 and Table S9), with 76.87% of predictions falling within 0.01 eV (Figure S6).

SSAL's extrapolation capability was validated on eight chemically diverse, state‐of‐the‐art electrocatalysts (Figure S7 and Table S10). Despite substantial compositional shifts, the predicted *R*_p_ values aligned with both experimental measurements and literature reports within their respective uncertainties. Collectively, these results establish SSAL as a robust and data‐efficient engine for forecasting experimental performance and accelerating exploration across complex electrocatalyst design spaces.

## 3 Mechanistic Insights From Data Mining

**FIGURE 3:**
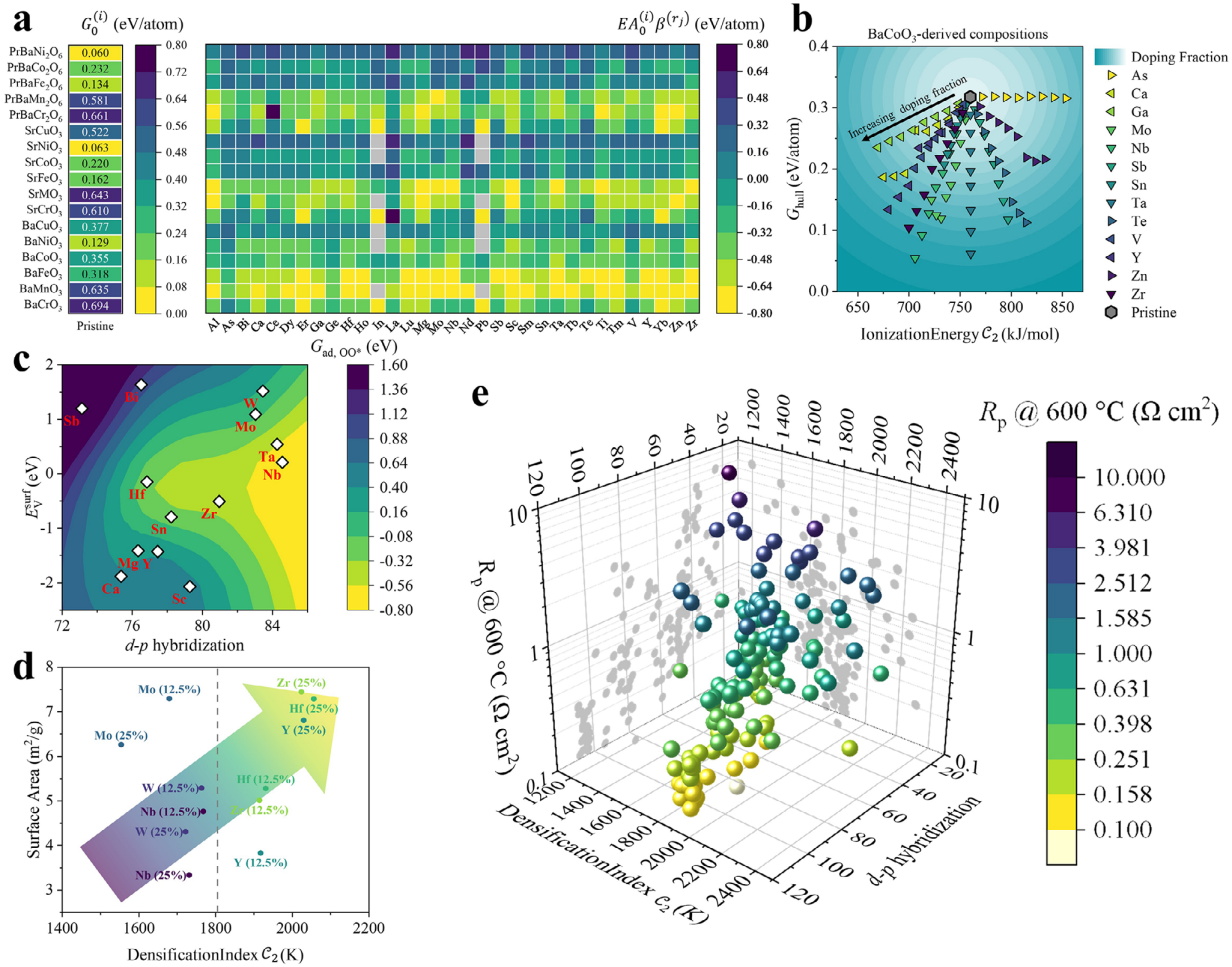
Structure–property performance insights. (a) Heat maps of critical factors embedded in the *G*_hull_ regression model. Left: baseline thermodynamic stability of the undoped parent lattice ($G_{0}^{\left(i\right)}$). Right: normalized modulation terms ($EA_{0}^{\left(i\right)}\beta^{\left(r_{j}\right)}$), highlighting the sensitivity of each composition to specific doping route. (b) Distribution of *G*_hull_ as a function of ionization energy (feature $C_{2}$) for representative BaCoO_3_‐derived compositions. (c) Contour map of *G*_ad, OO*_ plotted against *H_dp_* and $E_{V}^{\mathrm{surf}}$, illustrating the energetic balance governing surface reactivity. (d) Specific surface area, determined via BET measurements, as a function of the DensificationIndex (feature $C_{2}$) for representative BaCoO_3_‐derived compositions (doping fraction labeled). (e) Experimental *R*_p_ at 600°C as a function of *H_dp_* and DensificationIndex (feature $C_{2}$) across all experimentally synthesized candidates.

Thermodynamic stability was first analyzed to identify the primary features associated with variations in *G*_hull_. SHapley Additive exPlanations (SHAP) analysis revealed electron affinity as the most influential descriptor, accounting for 36% of model variance (mean SHAP = 0.12; Figure S8), substantially surpassing other features. Building upon this finding, we employed a mixed‐effects regression model to quantify route‐specific trends in *G*_hull_ across compositional space (Figure S9):

(1) $$G_{\mathrm{hull},\;ij}=G_{0}^{\left(i\right)}+EA_{0}^{\left(i\right)}\beta^{\left(r_{j}\right)}\alpha +\varepsilon_{ij}$$

where *G*_hull, *ij*_ is the DFT‐computed hull energy of composition *i* within doping route *r_j_*, as a function of doping fraction α. In this model, $G_{0}^{\left(i\right)}$ represents the inherent stability of the parent host lattice, $EA_{0}^{\left(i\right)}$ denotes its electron affinity (feature $C_{6}$; sixth linear combination derived from material featurization), and the residual term ε_*ij*_ captures variance attributable unmodeled factors, which is comparatively small within the fitted model. The term $EA_{0}^{\left(i\right)}\beta^{\left(r_{j}\right)}$ quantifies the deviation of the doped composition's *G*_hull_ from its pristine reference $G_{0}^{\left(i\right)}$, enabling a global description across diverse compositional categories. Figure 3a summarizes the $G_{0}^{\left(i\right)}$ values for 18 distinct perovskite parent host lattices, along with the normalized modulation terms $EA_{0}^{\left(i\right)}\beta^{\left(r_{j}\right)}$ for 33 associated doping routes. For example, pristine BaCoO_3_, with a ground‐state stability of $G_{0}^{\left(i\right)}$ = 0.355 eV/atom, serves as a moderately stable baseline. Doping with Nb or Ta yields $EA_{0}^{\left(i\right)}\beta^{\left(r_{j}\right)}$ values of −0.509 and −0.495 eV/atom, respectively, highlighting highly effective routes for reducing *G*_hull_, and consequently enhancing thermodynamic stability.

Going deeper, we investigated the physical interpretation of $\beta^{\left(r_{j}\right)}$ by isolating electron affinity effects in a subset of 337 doped compositions based on the BaCoO_3_ host lattice, where it functioned as a directional indicator (Figure S10). Notably, we observed distinct linear correlations between *G*_hull_ and dopant ionization energy (feature $C_{2}$; Figure 3b), with the sign and magnitude of the fitted slope reflecting the dopant's oxidation chemistry. These two descriptors exhibit a hierarchical relationship: electron affinity is strongly associated with broad thermodynamic stability trends across chemically heterogeneous systems, while ionization energy provides a secondary level of control by fine‐tuning redox accommodation within a given structural motif. Together, these parameters provide a quantitative framework for exploring trends in thermodynamic stability of perovskite materials.

On the other hand, ORR/OER electrocatalytic activity is strongly influenced by three variables—operating temperature, *H_dp_*, and densification resistance (DensificationIndex, feature $C_{2}$)—as revealed by feature‐attribution analysis, which assigns them SHAP values of 0.23, 0.12, 0.10, respectively (Figure S11). Given the well‐established Arrhenius relationship between temperature and electrocatalytic activity, operating temperature is expected to exert the strongest influence. Beyond temperature, *H_dp_* emerges as the most influential descriptor, and its mechanistic implications can be elucidated through fundamental analysis. DFT calculations on catalytic (001) BO_2_‐terminated surfaces of representative BaCoO_3_‐derived compositions (Supporting Note S5) reveal that a larger bulk *H_dp_* is correlated with stronger σ‐bonding interactions between Co 3d and O 2p orbitals. This correlation extends to the surface, as evidenced by increasingly negative ICOHP values for Co─O bonds and more delocalized electron density (Figures S12 and S13). Enhanced Co–O orbital overlap increases the surface concentration of the superoxide intermediate $(O_{I}^{q_{I}}\text{…}O_{\mathrm{II}}^{q_{\mathrm{II}}})*$ (hereafter abbreviated as OO*), which can facilitate the subsequent rate‐determining dissociative step,

(2)

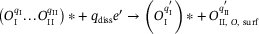

and is therefore consistent with enhanced ORR kinetics. By stabilizing lower‐energy transition states, this mechanism similarly promotes the OER process [13].

Through combined analysis incorporating the surface oxygen vacancy formation energy ($E_{V}^{\mathrm{surf}}$) (a descriptor excluded from the TSMS model due to its high computational cost), we identified a linear relationship between the Gibbs adsorption energy of the superoxide species (*G*_ad, OO*_) and *H_dp_*, whereas $E_{V}^{\mathrm{surf}}$ displayed a volcano‐type dependence (Figure 3c). Notably, doping with Nb or Ta produced near‐optimal *G*_ad, OO*_, characterized by both enhanced *H_dp_* and intermediate vacancy formation energies, which together are consistent with favorable surface adsorption of energetics.

Furthermore, DensificationIndex—a physically defined descriptor representing the weighted average melting point of oxide precursors—is recognized as another key factor, nearly matching the importance of *H_dp_*. This descriptor captures the inherent resistance to densification and strongly correlates with essential physical properties such as specific surface area, porosity, and tortuosity to mass transport, which otherwise require resource‐intensive BET measurements or detailed 3D reconstructions [38]. As shown in Figure 3d, higher DensificationIndex values correspond not only to increased surface areas and a greater density of accessible active sites for surface reactions, but also to reduced tortuosity for transport of reactants and products involved in electrode reactions—thereby enhancing electrocatalytic activity. Thus, this single physically grounded descriptor effectively captures extrinsic morphological features while still enabling accurate predictions of *R*_p_.

To complement SHAP‐based feature attribution with model‐independent validation, we performed partial‐dependence plot (PDP), individual conditional expectation (ICE), and counterfactual analyses (Supporting Note S2 and Figure S14). The PDPs show that the predicted *R*_p_ decreases overall with increasing *H_dp_* and DensificationIndex, while the corresponding ICE curves exhibit coherent trends across individual compositions. Counterfactual perturbation of ±1 standard deviation in either descriptor, with all other features held constant, yields directionally consistent changes in predicted log(*R*_p_) (Table S11), further corroborating their roles as key determinants of electrocatalytic activity.

Finally, plotting our 600°C electrocatalysts together with state‐of‐the‐art benchmarks in the *H_dp_*‐DensificationIndex plane (Figures 3e and S15) reveals consistent, positive correlations along both axes, showing that these two descriptors reliably rank *R*_p_ across chemically diverse materials. The fact that the benchmarks fall along the same trend line underscores the generality of the two‐descriptor map. It is important to note that these three dominant descriptors—temperature, *H_dp_*, and DensificationIndex—do not capture the full complexity of the system; the remaining features still contribute ∼50% for FSML and ∼62% for SSAL (Figure S16), reflecting the intrinsically multivariate nature of both thermodynamic stability and kinetic processes.

## 4 Large‐Scale Screening and Manifold Embedding

**FIGURE 4:**
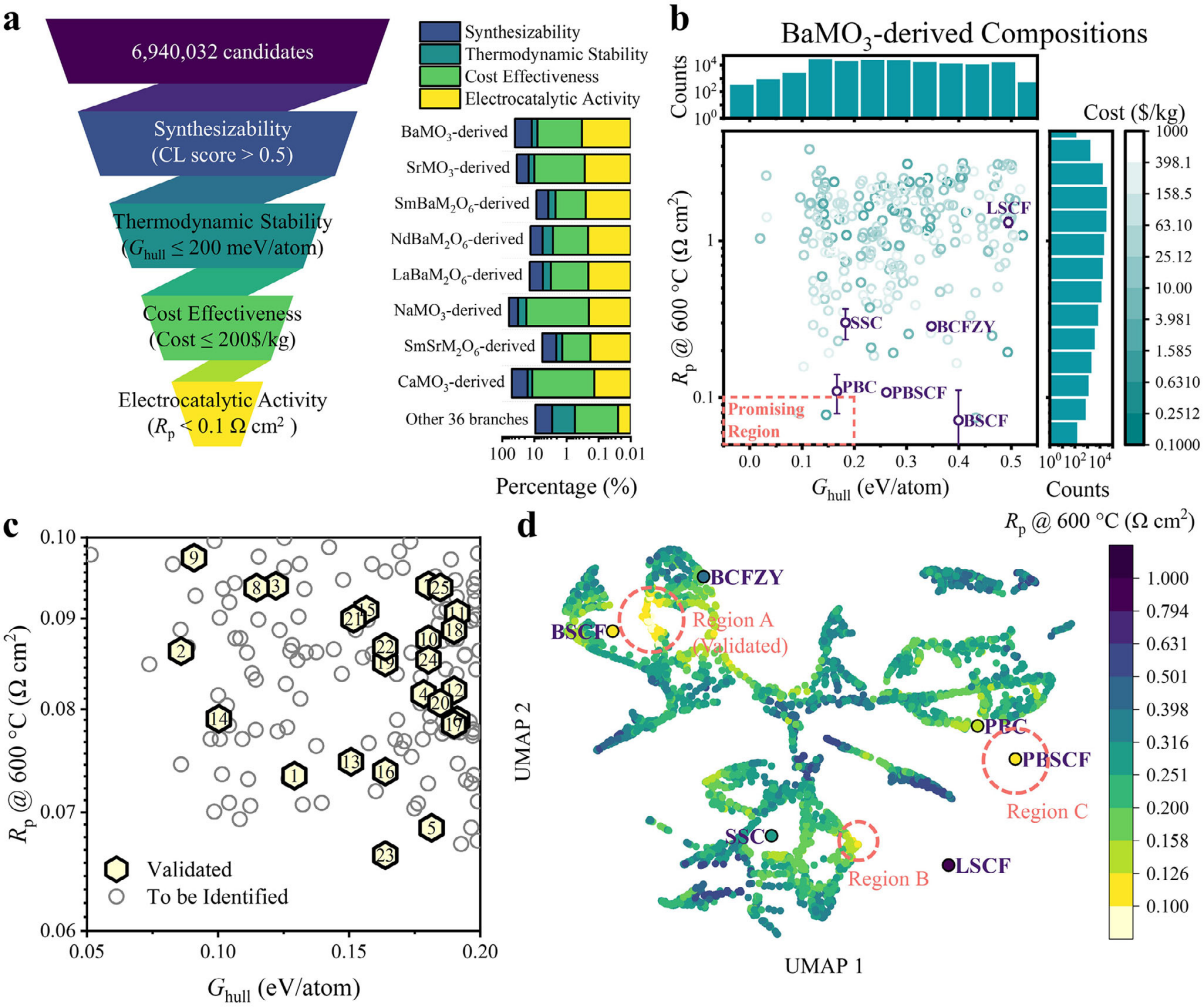
Application of TSMS to broader chemical space. (a) Funnel‐like screening of 6 940 032 candidates spanning 44 compositional branches, sequentially filtered by synthesizability, thermodynamic stability, cost‐effectiveness, and electrocatalytic activity. (b) Three‐dimensional distribution of BaMO_3_‐derived candidates, highlighting the identified promising region. Several state‐of‐the‐art materials are marked for comparison, including La_0.6_Sr_0.4_Co_0.2_Fe_0.8_O_3‐δ_ (LSCF), Ba_0.5_Sr_0.5_Co_0.8_Fe_0.2_O_3‐δ_ (BSCF), Sm_0.5_Sr_0.5_CoO_3‐δ_ (SSC), PrBaCo_2_O_5+δ_ (PBC), PrBa_0.5_Sr_0.5_Co_1.5_Fe_0.5_O_5+δ_ (PBSCF), and BaCo_0.4_Fe_0.4_Zr_0.1_Y_0.1_O_3‐δ_ (BCFZY). (c) Zoom‐in of the high‐performance region, with 25 experimentally validated candidates numerically labeled. (d) UMAP projection of the high‐dimensional chemical space based on *R*_p_‐related features, revealing distinct clustering of low‐*R*_p_ candidates and their proximity to known state‐of‐the‐art materials in the embedded manifold.

Leveraging the predictive power demonstrated in Stages I and II, we implemented a TSMS workflow to survey previously unexplored chemical space using minimal ground‐truth input (<0.1% of the total dataset). TSMS ranks four critical metrics—synthesizability (CL score) [39], thermodynamic stability (*G*_hull_), cost efficiency, and polarization resistance (*R*_p_, which is inversely proportional to electrocatalytic activity)—with efficiencies orders of magnitude higher than conventional DFT‐centered or purely experimental pipelines [40, 41]. Figure 4a illustrates the funnel‐like cascade applied to an unprecedented domain of 44 compositional branches (6 940 032 candidates in total; Supporting Note S1), from which only ∼0.062% (4287 cases) satisfy our stringent selection criteria. Among these, BaMO_3_ derivatives emerge as the leading family (Figure 4b), yielding 520 top‐ranked compositions. To date, 25 BaMO_3_‐derived compositions have already been synthesized and evaluated, all of which outperform state‐of‐the‐art benchmarks in *R*_p_ (Figure 4c and Table S12).

To visualize the feature landscape underlying the *R*_p_ predictor, we weighted each feature by its SHAP importance derived from SSAL and embedded the results into two dimensions using UMAP (Figure 4d) [42]. The resulting manifold reveals four promising regions. Region A—a broad performance plateau—contains both our validated BaMO_3_‐derived candidates and the state‐of‐the‐art material Ba_0.5_Sr_0.5_Co_0.8_Fe_0.2_O_3‐δ_ [43], indicating substantial compositional flexibility for further optimization beyond catalytic activity alone. Regions B exhibit steeper performance gradients and host several SrMO_3_ derivatives, while the unsampled Region C surrounds the high‐performing PrBa_0.5_Sr_0.5_Co_1.5_Fe_0.5_O_5+δ_ [44], highlighting a fertile space for future expansion. This performance‐oriented manifold not only uncovers latent similarities among disparate chemistries but also extends funnel‐like screening far beyond any single, predefined design space.

## 5 Experimental Validation and Device Integration

Experimental validation was performed on the most promising Nb‐ and Zr‐co‐doped BaCoO_3_ (BaCo_1‐x_(NbZr)_x_O_3‐δ_; BCNZ) series materials identified by TSMS, which consistently fulfilled all screening metrics across a broad doping window. A systematic examination of the BCNZ family pinpoints the origins of this superiority. Substituting either Nb or Zr lowers *G*_hull_ by tuning the ionization energies (Figure 3b), thereby enhancing thermodynamic stability. Nb simultaneously enhances *H_dp_* while maintaining a moderate $E_{V}^{\mathrm{surf}}$, which collectively regulate the Gibbs free energy of superoxide adsorption, *G*_ad, OO*_, as indicated in Figure 3c. This enables appropriate stabilization and sufficient surface population of superoxide intermediates on the oxygen electrode, thereby facilitating ORR/OER kinetics (Figure S17), consistent with trends observed across multiple perovskite families [45, 46]. Zr, meanwhile, increases the resistance to densification during thermal processing, as reflected by a higher DensificationIndex, thereby preserving a higher specific surface area under identical synthesis conditions (Figures S17 and S18). The synergistic action of these two dopants thus delivers concurrent gains in thermodynamic stability, microstructural robustness, and electrocatalytic activity. Through iterative experimental validation within the BCNZ family, BaCo_0.8_Nb_0.1_Zr_0.1_O_3‐δ_ (BCNZ20) was identified as the optimal composition (Figure S19), achieving the lowest *R*_p_ of 0.071 Ω cm^2^ at 600°C on symmetrical cells, among the lowest value reported to date for ORR/OER electrocatalysts (Figure S20). In addition, BCNZ20 displays a favorable apparent activation energy (*E*_a_) of 0.9 eV, reflecting the maximized synergistic benefits of Nb and Zr co‐doping.

**FIGURE 5:**
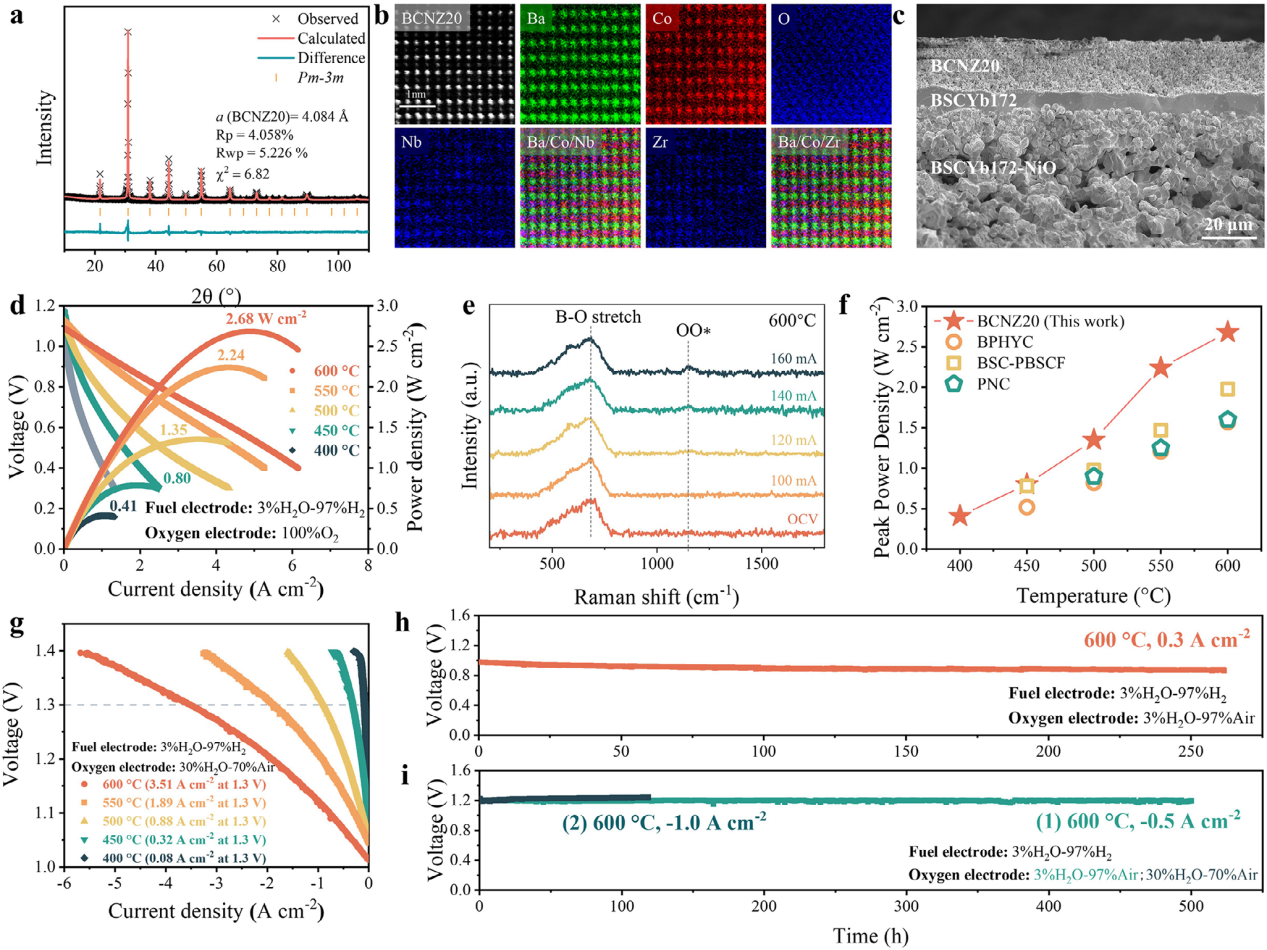
Structural, morphological, spectroscopic, and electrochemical characterization of P‐SOC single cells with BCNZ20 as the oxygen electrode. (a) X‐ray diffraction (XRD) patterns and Rietveld refinement of BCNZ20. (b) HAADF‐STEM image and corresponding EDS elemental maps of BCNZ20. (c) Cross‐sectional SEM image of the single cell with BCNZ20 as the oxygen electrode, BSCYb172 as the electrolyte, and BSCYb172‐NiO as the fuel electrode. (d) Representative *I*–*V*–*P* curves measured in fuel cell mode at 600‐400°C. (e) *Operando* Raman spectroscopy of BCNZ20 under fuel cell mode at 600°C. (f) Comparative analysis of peak power densities for P‐SOCs at 600°C–400°C, highlighting state‐of‐the‐art oxygen electrodes: Ba_0.9_Pr_0.1_Hf_0.1_Y_0.1_Co_0.8_O_3‐δ_ (BPHYC), Ba_0.62_Sr_0.38_CoO_3‐δ_‐Pr_1.44_Ba_0.11_Sr_0.45_Co_1.32_Fe_0.68_O_6‐δ_ (BSC‐PBSCF), and PrNi_0.7_Co_0.3_O_3‐δ_ (PNC). (g) Representative *I*–*V* curves measured in electrolysis cell mode at 600°C–400°C. (h and i) Long‐term durability of the BCNZ20|BSCYb172|BSCYb172‐NiO single cell at 600°C: (h) in fuel cell mode at a current density of 0.3 A cm^−2^, (i) in electrolysis cell mode at (i‐1) −0.5 A cm^−2^ (oxygen electrode exposed to 3%H_2_O–97%air) and (i‐2) −1.0 A cm^−2^ (oxygen electrode exposed to 30%H_2_O–70%air).

X‐ray diffraction (XRD) analysis (Figures 5a and S21) confirms BCNZ20 adopts a cubic perovskite structure with space group *Pm‐*3*m*, with no detectable impurity phases. This structural assignment is further supported by high‐resolution transmission electron microscopy (HRTEM) images combined with selected area electron diffraction (SAED) patterns obtained from low‐index crystallographic planes (Figures S22–S24). Atomic‐resolution high‐angle annular dark‐field scanning transmission electron microscopy (HAADF‐STEM), combined with energy‐dispersive x‐ray spectroscopy (EDS) elemental mapping and line‐scan analysis (Figures 5b and S25), provides direct evidence that Nb and Zr are uniformly incorporated into the perovskite lattice, substituting for Co at the B‐site, while Ba occupies the A‐site. Additionally, particle‐scale EDS mapping reveals a homogeneous elemental distribution throughout BCNZ20 (Figure S26). In situ x‐ray diffraction (XRD) provides a preliminary assessment of the structural stability of BCNZ20, showing that the perovskite phase is preserved across the full operating‐temperature range under both dry and humid air conditions (Figure S27 and Table S13).

Mechanistically, the exceptional performance of BCNZ20 originates from its fundamentally optimized reaction kinetics, as revealed by a combination of distribution of relaxation time (DRT) analysis and climbing‐image nudged elastic band (CI‐NEB) calculations. The favorable adsorption free energy of the superoxide intermediate (*G*_ad, OO*_ = −0.75 eV) ensures sufficient surface coverage of reactive superoxide species. This optimized adsorption facilitates an ultralow energy barrier for the subsequent rate‐determining step (RDS)—oxygen dissociation—which exhibits a record‐low transition‐state activation energy of only 0.57 eV (Equation 2; Figures S28–S30 and Supporting Note S6) [13, 34, 47]. Under humid conditions, a secondary proton‐involved RDS, peroxide hydrogenation, becomes operative yet remains kinetically facile with *E*_a_ = 0.38 eV (Figures S31 and S32).

Complementary experimental and computational analyses further demonstrate that BCNZ20 maintains this favorable kinetics through synergistic defect chemistry at both the surface and bulk levels. X‐ray photoelectron spectroscopy (XPS) reveals an abundance of surface hydroxide species that directly participate in the proton‐coupled reaction pathway (Figure S33). Thermogravimetric analysis (TGA) confirms substantial bulk hydration capacity (Figure S34), indicating that the lattice functions as an effective proton reservoir. Ab‐initio molecular dynamics (AIMD) simulations further reveal continuous proton‐transport pathways within the BCNZ20 bulk, enabling efficient proton migration and sustained replenishment of surface hydroxide species during operation (Movie S1).

To evaluate the device‐level applicability of the predicted candidates, we applied BCNZ20 as the oxygen electrode in a P‐SOC. After thoroughly investigating its fundamental properties and confirming its suitability as an oxygen electrode for P‐SOC applications (Figures S35–S41 and Supporting Note S7), we assembled a single cell with the configuration BCNZ20 | BaSn_0.1_Ce_0.7_Yb_0.2_O_3‐δ_(BSCYb172) | BSCYb172‐NiO, with the cross‐sectional SEM image shown in Figure 5c [35]. This cell employs a thin electrolyte layer, 5.8 µm in thickness, to minimize ohmic resistance and its impact on the overall cell performance. In fuel cell (FC) mode, the cell achieved peak power densities (PPDs) of 2.68, 2.24, 1.35, 0.80, and 0.41 W cm^−2^ at 600, 550, 500, 450, and 400°C, respectively (Figure 5d). To gain mechanistic insights into the oxygen electrode behavior under operating conditions, *operando* Raman spectroscopy was conducted in fuel cell mode at 600°C. As the output current of the P‐SOC single cell increased, a distinct Raman peak emerged at 1150 cm^−1^ (Figure 5e), corresponding to the accumulation of superoxide species (OO*) (Table S14) [48]. This observation provides direct evidence that superoxide intermediates accumulate on the electrode surface under operating conditions. It underscores the importance of achieving a more negative *G*_ad, OO*_ to enhance ORR/OER kinetics and is consistent with the DFT‐predicted mechanism in which superoxide formation precedes the rate‐determining dissociation step. Together with the CI‐NEB energy profiles, Bader charge analysis, and DRT‐based identification of the rate‐limiting process, these results collectively support the proposed reaction sequence. Notably, the PPD at 600°C exceeds the highest previously reported values for P‐SOCs by up to 35% (Figures 5f, S42 and S43; Tables S15 and S16) [2, 49] and is highly reproducible (Figure S44). In electrolysis cell (EC) mode, the same cell delivered current densities of 3.51, 1.89, 0.88, 0.32, and 0.08 A cm^−2^ at 600, 550, 500, 450, and 400°C, respectively (Figure 5g), under 30% water vapor at the oxygen electrode. These performance metrics place BCNZ20 among the highest‐performing oxygen electrodes reported to date (Figure S45). In addition, four other TSMS‐predicted candidates from Figure 4c consistently demonstrated excellent performance in both power‐generation and electrolysis modes (Figures S46–S49), underscoring the broad effectiveness and high precision of the screening workflow.

Long‐term durability was demonstrated under multiple operational modes. The P‐SOCs exhibited stable operation over 250 h in FC mode (0.3 A cm^−2^ at 600°C; Figure 5h), 500 h in EC mode (−0.5 A cm^−2^; Figure 5i‐1), and during repeated FC/EC cycling, with mode switching every two hours under a galvanostatic current of ± 0.3 A cm^−2^ at 600°C (Figure S50). Robust stability was further confirmed under more aggressive conditions (−1 A cm^−2^, 30% water vapor; Figure 5i‐2). SEM microstructural characterization and XRD analysis of the BCNZ20 oxygen electrode were conducted under three conditions: the as‐prepared state prior to electrochemical testing, after 250 h of operation in FC mode, and after 500 h of operation in EC mode (Figure S51). The BCNZ20 oxygen electrode retains its original perovskite phase and preserves its key microstructural features, with no evident grain coarsening, pore collapse, secondary phase formation, or other signs of degradation. These results confirm the strong microstructural and phase stability of the BCNZ20 oxygen electrode during prolonged electrochemical operation, consistent with its stable electrochemical performance.

Beyond proton‐conducting electrolytes, the generality of BCNZ20 was further validated in single cells employing an oxygen‐ion‐conducing electrolyte (Figure S52). In this configuration, BCNZ20 achieved a peak power density of 1.60 W cm^−2^ at 700°C and maintained stable fuel‐cell operation for 400 h at a current density of 0.3 A cm^−2^, highlighting its broad applicability as a high‐performance oxygen electrode across distinct electrolyte systems.

## 6 Conclusion and Prospect

By integrating physically informed high‐throughput methodologies with an active‐learning‐guided experimental feedback loop, we developed the TSMS framework to accurately predict key properties of ORR/OER electrocatalysts. These include thermodynamic stability (*G*_hull_), electronic structure descriptors (*p*‐band center, *d*‐band center, *H_dp_*), defect chemistry (*E*_V_, δ), and electrode polarization resistance (*R*_p_). Using SHAP‐based data mining, we uncovered hierarchical structure‐property relationships that reveal key descriptor dependencies governing both thermodynamic stability and ORR/OER kinetics across diverse chemical spaces. By combining a supervised machine learning‐based surrogate model (FSML) and an active learning strategy (SSAL), TSMS enables efficient exploration of ultra‐large chemical spaces, supports multi‐objective optimization, and guides targeted experimental validation. Among the experimentally validated candidates, five top‐performing compositions demonstrated record‐high electrocatalytic activity, long‐term durability, and cost effectiveness in P‐SOCs.

Together, these findings demonstrate the transformative potential of AI‐driven discovery frameworks to bridge first‐principles calculations, transferable machine learning, and accelerated experimentation via active learning. Using ORR/OER electrocatalysts as a case study, we systematically elucidated the fundamental electronic, atomistic, and kinetic factors underlying rational material design. More broadly, the strength of the TSMS framework lies in its hierarchical architecture, which enables seamless integration of multi‐fidelity data and physically informed embeddings. Its modular and extensible design allows incorporation of additional descriptors and design criteria, making it adaptable to a wide range of materials systems. Looking forward, this generalizable strategy offers a scalable pathway for accelerating materials discovery across diverse energy conversion and storage technologies—including fuel cells, batteries, solar cells, electrolyzers, and supercapacitors—ultimately advancing global efforts in decarbonization, resource efficiency, and sustainable energy deployment.

## Author Contributions

Xueyu Hu, Yucun Zhou, Haoyu Li, Zheyu Luo, Yuefeng Song, Minda Zou, Zhijun Liu, and Meilin Liu conceived the ideas for this study and designed the experiments. Xueyu Hu conducted high‐throughput computations and designed the machine learning model. Zhijun Liu, Yucun Zhou, and Zheyu Luo performed the fabrication and testing experiments of P‐SOC single cells. Haoyu Li, Yoojin Ahn, and Yuefeng Song performed the XPS analysis. Nai Shi performed the in situ XRD, TG, and TEC analysis. Yong Ding performed TEM analysis. Weining Wang and Weilin Zhang performed XRD, SEM, and BET analysis. Xueyu Hu, Doyeub Kim, Chanho Kim, and Nikhil Govindarajan synthesized and tested the oxygen electrocatalyst. Yuefeng Song conducted the HAADF‐STEM characterization and *operando* Raman analysis. Xueyu Hu, Zhijun Liu, Haoyu Li, and Meilin Liu wrote the manuscript. All authors have discussed and approved the final manuscript.

## Conflicts of Interest

The authors declare no conflicts of interest.

## Supporting information




**Supporting File 1**: adma74212‐sup‐0001‐SuppMat.docx.

**Supporting File 2**: adma74212‐sup‐0002‐MovieS1.mp4.

**Supporting File 3**: adma74212‐sup‐0003‐Data.zip.

## Data Availability

Data supporting the findings in the present work are available in the manuscript and Supporting Information. Coding information is available at https://github.com/XueyuHu/TSMS. Additional data are available from the corresponding authors upon reasonable request.
