# Supplementary material for: Mechanistically Interpretable Artificial Intelligence for Designing Oxygen Electrocatalysts
Source: Adv Mater. 2026 Jul 21;38(49):e74212. doi: 10.1002/adma.74212 (PMC13532494; doi:10.1002/adma.74212)
Supplement: Supplementary file 1 [file ADMA-38-e74212-s003.docx]

Supplementary Materials for

**Mechanistically Interpretable Artificial Intelligence for Designing Oxygen Electrocatalysts**

Xueyu Hu et al.

Corresponding author: Meilin Liu, meilin.liu@mse.gatech.edu, Yuefeng Song, songyf2014@dicp.ac.cn, Zhijun Liu, zhijun.liu@mse.gatech.edu

**Experimental Procedures**

**Standardized Experimental Procedure**

All ORR/OER electrocatalyst candidates were synthesized using the solid-state reaction (SSR) method. Stoichiometric amounts of metal oxides or metal carbonates (obtained from Aldrich Chemicals) were mixed with zirconia balls and ethanol, then ball milled at 400 rpm for 6 hours using a planetary ball mill. The dried precursor powder was collected, die-pressed into bulk samples, and calcinated at 1100 °C for 5 hours. The calcined bulk sample was crushed to produce coarse-grained catalyst powder, which was then refined by mixing with zirconia balls and ethanol and subjected to another round of ball milling at 400 rpm for 6 hours.

The refined catalyst powder was mixed with α-terpineol containing 6 wt.% ethyl cellulose to prepare a slurry at a ratio of 0.7:1. This slurry was then applied to both sides of the Sm_0.1_Ce_0.9_O_1.9_ (SDC) pellets using a screen-printing process. The coated substrates were subsequently calcined at 950 °C for 2 hours, forming a sandwich-structured catalyst/SDC/catalyst symmetrical cell, ready for electrochemical impedance spectroscopy (EIS) measurements.

The SDC pellets were created by uniaxial dry pressing of commercially available SDC powder (Fuel Cell Materials, US). After pressing, the green pellets were sintered at 1450°C for 5 hours, reaching a relative density of about 98%.

EIS measurements were performed using a Solartron 1255 HF frequency response analyzer connected with an EG&G PAR potentiostat model 273 A to determine the polarization resistance (*R*_p_) as a direct measure of ORR/OER electrocatalytic activity. Measurements were taken with an AC amplitude of 20 mV across a frequency range of 100 kHz to 0.01 Hz.

**Preparation of Electrolyte Powder**

BaZr_0.1_Ce_0.7_Y_0.1_Yb_0.1_O_3-δ_ (BZCYYb1711) and BaSn_0.1_Ce_0.7_Yb_0.2_O_3-δ_ (BSCYb172) electrolyte powders were synthesized using the SSR method. Stochiometric amounts of barium carbonate, zirconium oxide, cerium oxide, yttrium oxide, ytterbium oxide, and tin oxide powders (Aldrich Chemicals) were mixed by ball milling at 400 rpm for 4 hours in ethanol, followed by drying and calcination at 1100°C for 10 hours in air. The calcined powder was then ball-milled (400 rpm for 4 hours) and calcined again at 1100°C for 10 hours before further application.

**Fabrication of Single Cells**

The Ni-BSCYb172 fuel electrode-supported single cells were created using co-tape casting and co-sintering techniques. The BSCYb172 electrolyte layer, NiO-BSCYb172 functional layer, and NiO-BSCYy172 supporting layer were sequentially cast onto Mylar film, then co-sintered at 1400°C for 5 hours in air. The process for fabricating the oxygen electrode layer was identical to that used for the ORR/OER electrocatalysts.

**Characterization Methods**

Structural and surface characterization: X-ray diffraction (XRD) measurements were performed using a Panalytical X’Pert Pro Alpha-1 with CuKα1 radiation and an XCelerator detector in the 20-80° range. In-situ XRD measurements were conducted with a BRUKER D8 PHASER utilizing CuKα radiation. Experiments were carried out in two atmospheres, air and 3 vol% H2O-air (wet air), over a temperature range of 50-700°C with 50°C increments. Brunauer-Emmett-Teller (BET) surface area measurements quantified the specific surface area of the powders. X-ray photoelectron spectroscopy (XPS) was performed on a Thermo K Alpha system with a 180°, double-focusing hemispherical analyzer, a 128-channel detector, and a spot size of 10-400 μm.

Morphology and microstructure analysis: The cross-sectional microstructure and morphology of the cells were examined using a scanning electron microscope (SEM, Hitachi SU8010). The microstructure and morphology of the PBCHf010 electrodes were analyzed with transmission electron microscopy (TEM, JEOL 4000EX).

Thermal characterization: The dilatometer investigations (NETZSCH DIL402C, Germany) were carried out to determine the thermal expansion coefficient (TEC) using strip samples. Thermogravimetric analysis (TGA) was performed with the NETZSCH Jupiter STA449 F3. An initial heating to 700 °C in ambient air was used to remove adsorbed species, followed by stepwise cooling at 50 °C intervals with 40-minute equilibration periods. Next, the atmosphere was switched to wet air, and the temperature was ramped back up to 700 °C. Changes in weight at equivalent temperatures under both atmospheres provided insights into hydration performance.

Electrical and electrochemical measurements: The electrical conductivity and conductivity relaxation measurements were carried out using a four-probe technique (Keithley-2700). Electrochemical performance testing of single cells was done by mounting them on an alumina support tube with Ceramabond 552 (Aremco). The steam concentration was managed with a humidification system (Fuel Cell Technologies, Inc.). Durability monitoring was conducted using an Arbin multi-channel electrochemical testing system (MSTAT).

**SHapley Additive exPlanations (SHAP)**

The SHAP (Shapley) value of a feature $x_{j}$ is obtained by averaging its marginal contribution over all permutations of the full feature set $\mathcal{F=\{}x_{1},\ldots,x_{p}\}$:

$\phi_{j}\left( val \right)=\sum_{S\mathcal{\subseteq F/}\{x_{j}\}} \frac{\left| S \right|!\left( p-\left| S \right|-1 \right)!}{p!}\left( val\left( S\cup\left\{ x_{j} \right\} \right)-val\left( S \right) \right)$

Here, $S$ is any subset that excludes $x_{j}$, and $\left| S \right|$ is its cardinality. The function $val\left( S \right)$ denotes the model’s expected prediction when the features in $S$ are fixed while the remaining features are marginalized out. Thus, $val\left( S\cup\left\{ x_{j} \right\} \right)-val\left( S \right)$is the marginal contribution of $x_{j}$ to coalition $S$. Averaging this quantity over all coalitions yields a unique, fair attribution that satisfies the Shapley axioms of efficiency, symmetry, dummy, and additivity.

**Uniform manifold approximation and projection (UMAP)**

UMAP embeddings were generated in Python 3.11 with the umap-learn package (v0.5.5). Prior to dimensionality reduction, each descriptor $x_{k}$ was multiplied by its normalized SHAP importance $\tilde{w_{k}}=w_{k}/\sum_{i} w_{i}$ to yield a weighted feature matrix $\boldsymbol{X}^{*}=\boldsymbol{X}diag(\tilde{\boldsymbol{w}})$. UMAP was applied for two-dimensional projections using the following hyper-parameters: $n_{\mathrm{neighbors}}=15$, min_dist =0.1, metric = ‘elucidate’, init = ‘random’, and random_state = 0.

**Ab-initio molecular-dynamics (AIMD) simulation**

Ab initio molecular dynamics with an on-the-fly machine-learning force field (MLFF) was performed in VASP. Calculations employed the PAW method and the PBE exchange–correlation functional. A plane-wave cutoff of **400 eV**, Γ-point sampling for the 4a × 4a × 4a supercell, spin-polarization and an electronic convergence threshold of **1×10^-4^ eV** were employed. The simulated BCNZ20 supercell contained 64 Ba, 52 Co, 6 Nb, 6 Zr, 192 O, and 2 H atoms under three-dimensional periodic boundary conditions. All initial geometries were fully relaxed prior to dynamics.

On-the-fly training (10 ps): A canonical-ensemble (NVT) trajectory at 873 K, regulated by a Nosé–Hoover thermostat with a 0.5 fs time step, was used to iteratively build the MLFF. Configurations exceeding the model-uncertainty threshold were recomputed via DFT and incorporated into the training set, enabling incremental refinement of MLFF during the simulation.

Pre-equilibration: Following training, the finalized MLFF was frozen. The system was equilibrated sequentially at 300 K, 600 K, and 873 K for 2.5 ps each (NVT, 0.5 fs), ensuring removal of transients and establishment of a stable ensemble at the target temperature.

Production dynamics (250 ps): An NVT production run was subsequently performed at 873 K for 500,000 steps with a 0.5 fs time step (total 250 ps), with trajectories and log files recorded for subsequent analysis.

Supplementary Notes

**Note 1. Candidates Generation**

To construct the high-performance prototypes, we targeted the chemical space surrounding state-of-the-art (SOA) oxygen reduction/evolution (ORR and OER) electrocatalysts, including La_0.6_Sr_0.4_Co_0.2_Fe_0.8_O_3-δ_ (LSCF)^1^, Ba_0.5_Sr_0.5_Co_0.8_Fe_0.2_O_3-δ_ (BSCF)^2^, Sm_0.5_Sr_0.5_CoO_3-δ_ (SSC)^3^, BaCo_0.4_Fe_0.4_Zr_0.1_Y_0.1_O_3-δ_ (BCFZY)^4^, and PrBa_0.5_Sr_0.5_Co_1.5_Fe_0.5_O_5+δ_ (PBSCF)^5^. A focused library of 6,066 unique candidates was generated by systematically sampling their neighboring chemical spaces. This library incorporates variations in A-site elements (Ba, Sr, and PrBa) and B-site active elements (Cr, Mn, Fe, Co, Ni, Cu), yielding 18 distinct compositional branches. Each branch includes one pristine cubic perovskite (ABO_3_) and 336 ABO_3_-derived structures created through doping with 42 different elements at a resolution of 6.25%. To preserve sufficient electrocatalytic activity, the total doping content was limited to below 50%.

In the Large-Scale Screening section, we expand the applicability of the AI-driven Two-Stage Material Screening (TSMS) model, by implementing a dense chemical space sampling strategy across a much broader and previously underexplored compositional domain. For the A-site, 12 elements (Na, K, Rb, Cs, Mg, Ca, Sr, Ba, La, Pr, Nd, Sm) were selected. In addition, binary A-site combinations comprising one rare-earth element (La, Pr, Nd, and Sm) and one alkali or alkali earth metal (Na, K, Rb, Cs, Mg, Ca, Sr, or Ba) in a 1:1 ratio were considered, resulting in 12 single-element and 32 dual-element A-site configurations. For the B-site, six active elements (Cr, Mn, Fe, Co, Ni, Cu) were chosen along with 31 dopants (Al, As, Bi, Ce, Dy, Er, Eu, Ga, Gd, Ge, Hf, Ho, In, Lu, Mo, Nb, Pb, Sb, Sc, Sn, Ta, Tb, Te, Ti, Tm, V, W, Y, Yb, Zn, Zr). Both single and co-doping strategies were applied, while ensuring that the fraction of B-site active elements remained ≥ 0.5. This strategy led to the construction of a vast library of 6,940,032 distinct candidates, enabling near-continuous sampling of an expansive chemical space and significantly enhancing the versatility of the TSMS framework for generalizable material discovery.

**Note 2. First-stage machine learning (FSML)**

A. Material Featurization

The FSML process begins with materials featurization, wherein atomic, bonding, and structural attributes are systematically extracted as input descriptors:

A1. Atomic attributes

Elemental type (Z) and stoichiometry (N) for the major A-site, A-site dopant, B-site active, B-site dopant elements. (8 features)

Fundamental physicochemical properties, including electron affinity (EA), oxidation state (OS), melting point (T_m_), boiling point (T_b_), atomic mass (M), elemental density (ρ), ionization energy (I), electronegativity (χ), and chemical potential of the oxide (μ), were derived through twelve distinct linear combinations ($\mathcal{C}$): (108 features)

($\mathcal{C}_{1}$) Weighted average of the property for A-site elements, based on their fractions.

($\mathcal{C}_{2}$) Weighted average of the property for B-site elements, based on their fractions.

($\mathcal{C}_{3}$) Weighted average of the property for A-site dopant elements, based on their fractions.

($\mathcal{C}_{4}$) Weighted average of the property for B-site dopant elements, based on their fractions.

($\mathcal{C}_{5}$) Weighted average of the property for A-site major elements, based on their fractions.

($\mathcal{C}_{6}$) Weighted average of the property for B-site active elements, based on their fractions.

($\mathcal{C}_{7}$) Sum of ($\mathcal{C}_{5}$) and ($\mathcal{C}_{6}$).

($\mathcal{C}_{8}$) Ratio of ($\mathcal{C}_{1}$) to ($\mathcal{C}_{2}$).

($\mathcal{C}_{9}$) Ratio of ($\mathcal{C}_{5}$) to ($\mathcal{C}_{6}$).

($\mathcal{C}_{10}$) Ratio of ($\mathcal{C}_{3}$) to ($\mathcal{C}_{1}$).

($\mathcal{C}_{11}$) Ratio of ($\mathcal{C}_{4}$) to ($\mathcal{C}_{2}$).

($\mathcal{C}_{12}$) Sum of ($\mathcal{C}_{3}$) and ($\mathcal{C}_{4}$) divided by the sum of ($\mathcal{C}_{5}$) and ($\mathcal{C}_{6}$).

A2. Bonding attributes

Bonding-related descriptors include Shannon ionic radii (r), DFT-calculated bond length ($d_{\mathrm{DFT}}$), the DensificationIndex (DI), and Crystal Orbital Hamilton Population (COHP) values. These features were further expanded using twelve distinct linear combinations ($\mathcal{C}_{1},\ldots,\mathcal{C}_{12}$), consistent with the atomic descriptor treatment. (48 features)

A3. Structural attributes

Four structural descriptors were included, capturing geometric and stoichiometric characteristics of perovskite structures: Two types of tolerance factors (Goldschmidt’s t and modified *τ*) ^6^, the octahedral factor (*o*), and the oxygen non-stoichiometry (*δ*).(4 features)

*t* is defined as:

$t=\frac{r_{A}+r_{O}}{\sqrt{2}(r_{B}+r_{O})}$	(S1)

where $r_{A}$, $r_{B}$, and $r_{O}$ are the weighted average radii of A-site and B-site cations, and the radius of oxygen ions, respectively: $r_{A}= \sum_{i} r_{A_{i}}*f_{A_{i}},$ $R_{A}= \sum_{i} r_{B_{i}}*f_{B_{i}}$.

*τ* is defined as:

$\tau=\frac{r_{O}}{r_{B}}-n_{A}(n_{A}-\frac{r_{A}/r_{B}}{ln(r_{A}/r_{B})})$	(S2)

where $n_{A}$ is the weighted average oxidization state of A-site cations: $n_{A}= \sum_{i} n_{A_{i}}*f_{A_{i}}$.

*o* is defined as:

$o=\frac{r_{B}}{r_{O}}$	(S3)

*δ* is learned via machine learning regression based on the dataset constructed from Li et al’s work ^7^.

B. Training

Following the materials featurization, a rigorous engineering process was employed to prepare the input data for the FSML model. To mitigate the influence of extreme outliers, the top and bottom 0.5% of feature values across all samples (1% in total) were clipped to their respective 0.5th and 99.5th percentile values. This treatment effectively reduced the impact of distant during model training. All features were subsequently normalized to ensure compatibility across scales.

The dataset was randomly split into training and test sets at a 4:1 ratio. A detailed breakdown of the chemical composition of the training and test sets, including the number of parent lattices, B-site branches, dopant elements, dopant pairs, and dopant routes, is provided in Table S3. Multiple machine learning algorithms were evaluated, including Least Absolute Shrinkage and Selection Operator (LASSO), Support Vector Regression (SVR), Random Forest (RF), XGBoost (Extreme Gradient Boosting), and Artificial Neural Networks (ANN). Five-fold cross-validation was conducted on the training assess the performance of each algorithm.

Model performance was quantified using the root mean square errors (RMSE) and the coefficient of determination (*R*^2^), defined as:

$RMSE=\sqrt{\frac{1}{n}\sum_{i=1}^{n} {(y_{i}-\hat{y_{i}})}^{2}}$	(S4)

$R^{2}=1-\frac{\sum_{i=1}^{n} {(y_{i}-\hat{y_{i}})}^{2}}{\sum_{i=1}^{n} {(y_{i}-\bar{y})}^{2}}$	(S5)

where $y_{i}$ is the actual value, $\hat{y_{i}}$ is the predicted value, and $\bar{y}$ is the mean of the actual values. Among all algorithms, XGBoost yielded the best performance and was selected for subsequent use. Hyperparameters of XGBoost model were further optimized to enhance accuracy and generalizability (Table S1).

C. Generalizability

To assess whether the FSML model captures transferable physicochemical trends beyond route-specific patterns, we performed leave-one-route-out (LORO) cross-validation. Here, a “dopant route” is defined as a unique combination of the parent host lattice (A-site element) and the B-site dopant identity. For example, Ba-Co-Nb represents all BaCoO_3_-derived compositions doped with Nb, encompassing doping fractions from 6.25% to 50%. Compositions within the same route share the same elemental combination and differ only in dopant concentration, rendering them chemically highly similar.

In the LORO scheme, all compositions belonging to a given dopant route are entirely excluded from the training set and used as the test set. The model is then trained on the remaining routes and subsequently evaluated on the held-out route. This procedure is repeated for each of the 337 dopant routes, and the predictions are aggregated across all held-out samples to compute overall performance metrics. Unlike random splitting, where compositions from the same route with different doping fractions may appear in both training and test sets, LORO ensures that the model has never encountered any composition from the test route during training. This setup provides a stringent assessment of the model’s ability to generalize across dopant chemistries.

To extend the applicability of the FSML model to LaMO_3_-derived candidates, a transfer learning strategy was employed. The pre-trained FSML model, originally trained on the full set of 6,066 candidates, served as the initialization point. A 1% subset of the new LaMO_3_ dataset was selected for fine-tuning. During this retraining phase, model hyperparameters were adjusted to improve learning efficiency and reduce overfitting: the maximum tree depth was increased to 5, the learning rate was decreased to 0.01, and the ensemble size was expanded to 500 trees.

To evaluate the generalizability of the FSML model, we generated an out-of-distribution dataset by selecting a completely different A-site element, La, which was absent from the original 6,066 candidates. The selection criteria for B-site elements remained consistent, resulting in the creation of 2022 new LaMO_3_-derived candidates. High-throughput calculations were performed on these candidates to obtain their ground truth computational information.

D. Partial Dependence, ICE, and Counterfactual Analysis

To further validate the descriptor–performance relationships identified by the SHAP analysis, we performed partial-dependence plot (PDP), individual conditional expectation (ICE), and counterfactual analyses using the trained SSAL model.

For a given feature $x_{j}$, the partial-dependence function is defined as:

$$\hat{f}_{\mathrm{PDP}}\left( x_{j} \right)=\frac{1}{N}\sum_{i=1}^{N} \hat{f}(x_{j},x_{i,-j})$$

where $\hat{f}$ is the trained model,$x_{i,-j}$ denotes all features of sample i except $x_{j}$, and $N$ is the total number of samples. The PDP marginalizes over the remaining features to isolate the average effect of $x_{j}$ on the predicted output. A uniform grid of 50 equally spaced values spanning the observed range of $x_{j}$ was used.

Each ICE curve traces the predicted output for a single sample $i$ as $x_{j}$ varies across the same grid, with all other features held at their observed values:

$$\hat{f}_{\mathrm{ICE}}\left( x_{j} \right)=\hat{f}(x_{j},x_{i,-j})$$

The PDP is the pointwise average of all ICE curves. Comparing individual ICE curves against the PDP reveals whether the effect of $x_{j}$ is consistent across samples or modulated by interactions with other features.

For the counterfactual analysis, we perturbed a single feature (*H_dp_* or DensificationIndex) by ±1 standard deviation of its distribution across the full dataset for each sample in the test set, while holding all other features at their original values. The resulting change in predicted log(*R*_p_) quantifies the sensitivity of the model output to each descriptor at the individual-sample level.

**Note 3. ORR/OER electrocatalytic descriptors**

High-throughput density functional theory (DFT) calculations were conducted to generate computational descriptors and parameters. These calculations were performed using the Vienna ab initio simulation package (VASP)^8, 9^. The generalized gradient approximation (GGA) with Perdew-Butke-Ernzerhof (PBE) functional was employed to account for exchange-correlation effects in the Kohn-Sham equations^10^. Pseudopotentials and effective Hubbard U values were selected according to the Material Project database^11^ (see Table S2 for details). The energy cutoff and convergence criteria were set as 400 eV and 4×10^-3^ eV, respectively. The RMM-DIIS algorithm and conjugate gradient method were utilized for electronic and ionic optimization, respectively. A supercell of dimensions $2\sqrt{2}a\times2\sqrt{2}a\times2a$ was employed, enabling a doping fraction resolution of 6.25%. A Γ-centered k-point grid equivalent to a density of 150 k-points/Å^3^ in reciprocal space was employed.

To capture finite-temperature configurational disorder, Molecular Dynamics-Monte Carlo (MD-MC) simulations were initially performed for each candidate using the Large-scale Atomic/Molecular Massively Parallel Simulator (LAMMPS) in combination with the M3Gnet potential^12, 13^. These simulations informed the elemental distribution and served as input for subsequent structural relaxations and static DFT calculations. For each relaxed structure, the density of states (DOS) was computed with a doubled k-point density to enhance resolution.

The following ORR/OER-relevant descriptors were calculated:

1. Gibbs free energy above the convex hull (*G*_hull_) was determined using the computed energy from static calculation, cross-referenced with the Materials Project database. Temperature and atmosphere corrections were applied to calculate the *G*_hull­_ values under P-SOCs operation conditions (T = 600 °C, *p*(O_2_) = 0.21 atm)^14^.

2. The oxygen *p*-band center ($\bar{O}_{2p}$) is defined as:

$\bar{O}_{2p}=\frac{\int_{-\infty}^{\infty} E\cdot D_{O_{2p}}(E)dE}{\int_{-\infty}^{\infty} D_{O_{2p}}(E)dE}-E_{Fermi}$	(S6)

where $E$ is the electron energy, $D_{O_{2p}}(E)$ is the DOS projected onto the oxygen 2*p*-orbitals, and $E_{Fermi}$ is the Fermi energy.

3. The *d*-band center of the active element M ($\bar{M}_{3d}$) was defined as:

$\bar{M}_{3d}=\frac{\int_{-\infty}^{\infty} E\cdot D_{M_{3d}}(E)dE}{\int_{-\infty}^{\infty} D_{M_{3d}}(E)dE}-E_{Fermi}$	(S7)

where $D_{M_{3d}}(E)$ represents the DOS projected onto the 3*d-*orbitals of M.

4. The oxygen vacancy formation energy (*E*_V_) was evaluated by removing one oxygen atom and calculating the defective structure, with the vacancy positioned near the dopant to highlight its influence. *E*_V_ is defined as

$E_{V}=E_{V_{O}^{\cdot\cdot}}+\frac{1}{2}E(O_{2})-E_{P}$	(S8)

where $E_{V_{O}^{\cdot\cdot}}$ is the total energy of the defective bulk with a single oxygen vacancy, $E_{P}$ is the total energy of the pristine bulk, and $E(O_{2})$ is the total energy of an isolated oxygen molecule.

5. The hybridization of the *d-* and *p-*bands (*H_dp_*) was quantified by the overlap extent of their DOS plots:

$H_{dp}=\int_{-\infty}^{+\infty} min(D_{O_{2p}}^{up}\left( E \right),D_{M_{3d}}^{up}\left( E \right))dE+\int_{-\infty}^{+\infty} min(D_{O_{2p}}^{down}\left( E \right),D_{M_{3d}}^{down}\left( E \right))dE$	(S9)

where $D_{O_{2p}}^{up}\left( E \right)$ and $D_{M_{3d}}^{up}\left( E \right)$ are the spin-up DOS projections onto the oxygen 2*p-* and active element 3*d*-orbitals, respectively, and $D_{O_{2p}}^{down}\left( E \right)$ and $D_{M_{3d}}^{down}\left( E \right)$ are the spin-down counterparts.

6. Twelve additional descriptors were extracted from relaxed structures, including lattice constants ($a, b, c, \alpha, \beta, \gamma$), unit cell volume, free volume, structural distortion upon oxygen vacancy formation, space group, point group, and the number of symmetry operations. These parameters were incorporated into the machine learning models to enhance predictive accuracy and physical interpretability.

**Note 4. Second-stage active learning (SSAL)**

A. Multi-objective optimization

Multi-objective optimization (MOOP) was employed to construct the initial dataset for SSAL training. The process began with a preliminary screening of the multi-dimensional descriptor space defined by the attribute sets {$\bar{O}_{2p}$, $\bar{M}_{3d}$, *E*_V_, δ, *H_dp_*}, as identified in Stage Ⅰ. A global weighted-sum objective function was applied to the normalized descriptor space^15^:

$f\left( x \right)=\sum_{i} w_{i}\cdot x_{i}$	(S10)

Equal weights were assigned to all five descriptors ($w_{i}$ = 1/5 for all $i$), as no experimental *R*_p_ data were available at this stage to justify differential weighting. Each descriptor was min–max normalized to [0, 1] prior to aggregation, with the normalization direction defined such that lower values of the composite score $S$ correspond to more favorable predicted electrocatalytic activity. This equal-weight formulation ensures that the MOOP-selected seed set broadly samples the multi-dimensional descriptor space without introducing prior bias toward any single physicochemical property. MOOP selected the top 1% candidates (60 entries) predicted to exhibit superior electrocatalytic activity. These candidates were then subjected to standardized experiments to obtain their *R*_p_ at 600, 650, and 700 °C, along with the corresponding *E*_a_, yielding a total of 240 experimental entries.

B. Active learning

The active learning framework was built on a Gaussian process (GP) surrogate model, used exclusively for uncertainty-aware acquisition and sequential experimental selection. In each iteration, the GP model provided both predictive mean and posterior uncertainty estimates for unexplored candidates, enabling principled uncertainty quantification.

In parallel, XGBoost regression models were employed separately for post hoc performance evaluation, feature importance analysis, and final high-accuracy prediction after data acquisition.

The initial dataset comprised 180 experimentally measured *R*_p_ values and 60 *E*_a_ values. The data were split into training and test sets in a 4:1 ratio, and five-fold cross-validation was used for model evaluation. To improve robustness and reduce overfitting due to limited data size, an ensemble of 50 independent GP models was trained using different random seeds, with variations in data shuffling, sampling, and initialization. This ensemble collectively formed the first-generation sequential self-adaptive learning (SSAL) predictor.

Bayesian optimization was then employed to iteratively propose new experiments. Specifically, the Expected Improvement (EI) acquisition function was adopted to balance exploitation of candidates with low predicted *R*_p_ values and exploration of regions with high predictive uncertainty. For a candidate 𝑥, the EI is defined as:

$\mathrm{EI}\left( x \right)\mathbb{=E}[max(0, f_{\mathrm{best}}-f(x))]$	(S11)

Assuming the GP model yields a predictive distribution $f\left( x \right)\mathcal{\sim N(}\mu\left( x \right),\sigma^{2}(x))$, the EI can be expressed analytically as:

$\mathrm{EI}\left( x \right)=\left( f_{\mathrm{best}}-\mu\left( x \right) \right)\Phi\left( Z \right)+\sigma\left( x \right)\phi\left( Z \right), Z=\frac{f_{\mathrm{best}}-\mu\left( x \right)}{\sigma(x)}$	(S12)

Here $\Phi\left( Z \right)$ and $\phi(Z)$ denote the cumulative distribution function and probability density function of the standard normal distribution, respectively. The term $f_{\mathrm{best}}$ represents the best experimentally observed *R*_p_ (or *E*_a_) value to date. Uncertainty estimates were derived directly from the GP posterior variance, rather than relying on ensemble-based or heuristic approximations.

In each learning round, the GP-based SSAL model evaluated all remaining candidates in the pool (6066 entries), and those ranked within the top 0.2% according to the EI score were selected for experimental synthesis and validation. The newly acquired experimental data were then incorporated into the training set, and the model was retrained to form the next-generation SSAL predictor. This iterative procedure was repeated for seven rounds.

C. Quantitative Convergence Analysis

To rigorously define the convergence of the SSAL process, we evaluated two complementary

quantitative criteria at each acquisition round, reflecting the dual objectives of the active learning loop: discovering candidates with the lowest possible *R*_p_ (exploitation) and building a reliable predictive model across the broader chemical space (exploration).

**Criterion 1: Model accuracy saturation.** As shown in Table S7, as additional experimental data were progressively incorporated into the training set, the RMSE for log(*R*_p_) decreased and the corresponding *R*^2^ saturated above 0.95 across all rounds, consistent with the learning curve in Fig. S4. This indicates that additional data no longer yield meaningful improvement in predictive accuracy.

**Criterion 2: Candidate ranking stability.** At each round, we applied the updated model to

rank all candidates in the screening pool and computed the Spearman rank correlation coefficient

(ρ) between consecutive rounds. As shown in Table S7, ρ increased from 0.870 (Round 1-2) to 0.950-0.971 in later rounds (Rounds 4-7), indicating near-identical ordering of the entire candidate pool. The top-10 overlap between consecutive rounds remains consistently high (≥8/10), further confirming that the model’s identification of the most promising region has stabilized.

**Note 5 Data-mining details**

To elucidate the correlation between *H_dp_* and ORR/OER electrocatalytic activity, detailed DFT calculations were performed on 13 BaCoO_3_-derived candidates with enhanced energy cutoff (520 eV) and stricter convergence criteria (1×10^-5^ eV). Structural relaxation was carried out until the residual force on each atom was below 0.02 eV Å^-1^.

The systems studied were BaCo_0.875_M_0.125_O_3_ compositions, where the dopant M includes: Bi, Ca, Hf, Mg, Mo, Nb, Sb, Sc, Sn, Ta, W, Y, Zr. Each bulk model was constructed using a $2\times2\times2$ supercell, comprising 8 Ba, 7 Co, 1 M, and 24 O atoms. To capture the surface properties relevant to ORR/OER, the (001) BO_2_-terminated surface, previously identified as the most catalytic active facet^16^, was cleaved from the bulk. An eight-layer slab structure ($2\times2\times4$ supercell) was constructed, with the bottom four layers fixed during relaxation. A vacuum layer of 15 Å was added along the z-direction to prevent interactions between periodic images, and dipole corrections were applied to eliminate spurious electric fields. The bonding strength between the surface cobalt atoms and their neighboring oxygen atoms was quantified using the Integrated COHP values, computed using the LOBSTER code^17^.

The formation of a surface oxygen vacancy was modeled as spontaneous defect reaction:

$O_{O,\mathrm{surf}}^{\times}+2h^{\cdot}\to V_{O, surf}^{\cdot\cdot}+\frac{1}{2}O_{2}$	(S13)

where $O_{O,\mathrm{surf}}^{\times}$ denotes a lattice oxygen atom at the surface, $V_{O, surf}^{\cdot\cdot}$ is the surface oxygen vacancy, and $O_{2}$ is an isolated gas-phase oxygen molecule. The corresponding vacancy formation energy is given by:

$E_{V}^{\mathrm{surf}}=E_{V_{O, surf}^{\cdot\cdot}}+\frac{1}{2}E_{O_{2}}-E_{O_{O,\mathrm{surf}}^{\times}}$	(S14)

The adsorption of a gas-phase oxygen molecule at the vacancy site was modeled as:

$V_{O, surf}^{\cdot\cdot}+O_{2,gas}+q_{\mathrm{ads}}e^{'}\to\left( O_{Ⅰ}^{q_{Ⅰ}}\ldots O_{Ⅱ}^{q_{Ⅱ}} \right)*$	(S15)

where $\left( O_{Ⅰ}^{q_{Ⅰ}}\ldots O_{Ⅱ}^{q_{Ⅱ}} \right)*$ represents the adsorbed superoxide species. The adsorption energy of the superoxide (*E*_ad, OO*_) was calculated as:

$E_{\mathrm{ad},OO*}=E_{\left( O_{Ⅰ}^{q_{Ⅰ}}\ldots O_{Ⅱ}^{q_{Ⅱ}} \right)*}-E_{V_{O, surf}^{\cdot\cdot}}-E\left( O_{2,gas} \right)$	(S16)

Temperature correction were applied to obtain the Gibbs free energy of adsorption (*G*_ad, OO*_), incorporating vibrational entropy and zero-point energy contributions of the adsorbed oxygen species. Bader charge analysis was performed to quantify charge redistribution and electron delocalization upon vacancy formation and superoxide adsorption, providing insights into surface reactivity and charge transfer characteristics.

**Note 6. ORR mechanism**

To delineate the detailed energy profile of the ORR on BCNZ20, comprehensive DFT calculations were performed ^14^.

Conventional ORR Pathway: As shown in Fig. S28, the conventional ORR mechanism begins with the presence of an inherent surface oxygen vacancy ($V_{O, surf}^{\cdot\cdot}$), which creates an unsaturated coordination environment around the adjacent cobalt atom. This vacancy facilitates the adsorption of a gas-phase oxygen molecule, forming a surface-bound superoxide species,

Step 1:	$V_{O, surf}^{\cdot\cdot}+O_{2,gas}+q_{\mathrm{ads}}e^{'}\to\left( O_{Ⅰ}^{q_{Ⅰ}}\ldots O_{Ⅱ}^{q_{Ⅱ}} \right)*$	(S15)

This process characterized by an adsorption energy of -0.75 eV on the BO_2_-terminated (001) BCNZ20 surface. Bader charge analysis reveals partial charges of $q_{Ⅰ}$= 0.31 e and $q_{Ⅱ}$= 0.41 e for the two oxygen atoms, $O_{Ⅰ}$ and $O_{Ⅱ}$, respectively, as illustrated in Fig. S28.

The subsequent dissociation of the superoxide, identified as the rate-determining step (Fig. S29), has a low activation energy barrier of 0.57 eV:

Step 2:	$\left( O_{Ⅰ}^{q_{Ⅰ}}\ldots O_{Ⅱ}^{q_{Ⅱ}} \right)*+q_{\mathrm{diss}}e^{'}\to\left( O_{Ⅰ}^{q_{Ⅰ}^{'}} \right)*+O_{Ⅱ, O, surf}^{q_{Ⅱ}^{'}}$	(S17)

After dissociation, the charge transfer increases $q_{Ⅰ}^{'}$ = 0.40e and $q_{Ⅱ}^{'}$ = 0.78 e, indicating substantial charge transfer, consistent with DRT analysis. The surface bond species is identified as a peroxide species $(O_{Ⅰ}^{q_{Ⅰ}^{'}})*$. Oxygen transport follows as the dissociated $O_{Ⅱ}$ atom mitigates into the bulk oxygen site, regenerating a surface vacancy:

Step 3-1:	$O_{Ⅱ, O, surf}^{q_{Ⅱ}^{'}}+V_{O,bulk}^{\cdot\cdot}+q_{\mathrm{trans}}e^{'}\to V_{O, surf}^{\cdot\cdot}+O_{O,bulk}^{\times}$	(S18)

The remaining peroxide species $(O_{Ⅰ}^{q_{Ⅰ}^{'}})*$ subsequently undergoes an incorporation reaction step into the surface lattice:

Step 4-1:	$\left( O_{Ⅰ}^{q_{Ⅰ}^{'}} \right)*+V_{O, surf}^{\cdot\cdot}+q_{\mathrm{incorp}}e^{'}\to O_{Ⅰ, O, surf}^{q_{Ⅰ}^{''}}$	(S19)

During this process, the Bader charge of $O_{Ⅰ}$ increases from $q_{Ⅰ}^{'}=$ 0.52 e to $q_{Ⅰ}^{''}=$ 0.97 e, with an *E*_a_ of 0.49 eV. This closes one complete ORR cycle with the re-formation of a surface oxygen vacancy (Fig. S30).

Proton-involved ORR: The PI-ORR pathway shares the same initial adsorption and dissociation steps as the conventional mechanism (Eqs. S15 and S17) but diverges by incorporating surface protons (${OH}_{O,surf}^{\cdot}$) (Figs. S31 and S32).

The first hydrogenation step converts the peroxide species into a surface hydroxide $\left( {OH}_{Ⅰ}^{q_{Ⅰ}^{'''}} \right)*$,

Step 3-2:	$\left( O_{Ⅰ}^{q_{Ⅰ}^{'}} \right)*\ldots{OH}_{O,surf}^{\cdot}+q_{hydro1}e^{'}\to\left( {OH}_{Ⅰ}^{q_{Ⅰ}^{'''}} \right)*+O_{O,surf}^{\times}$	(S20)

This step proceeds with a low *E*_a_ of 0.38 eV, significantly lower than the 0.49 eV required for the conventional incorporation step (Eq. S19).

The second hydrogenation reaction leads to the water formation and surface regeneration,

Step 4-2:	$\left( {OH}_{Ⅰ}^{q_{Ⅰ}^{'''}} \right)*\ldots{OH}_{O,surf}^{\cdot}+q_{hydro2}e^{'}\to{H_{2}O}_{\mathrm{gas}}+O_{O,surf}^{\times}$	(S21)

This reaction is thermodynamically favorable, with a water desorption energy of -1.22 eV. The PI-ORR mechanism thus offers an energetically more accessible pathway for ORR at low temperatures, with proton-mediated steps facilitating surface regeneration.

By stabilizing the lower-energy transition states, this mechanism similarly promotes the OER process.

**Note 7. Fundamental properties of BCNZ20**

The key fundamental properties of BCNZ20 were systematically evaluated from five critical perspectives: (1) electrical conductivity (σ), (2) oxygen transport properties (*k*_Chem_, *D*_Chem_), (3) chemical compatibility, (4) thermal expansion coefficient (TEC), and (5) steam-enhanced ORR/OER kinetics.

(1) Electrical Conductivity: A dense strip sample of BCNZ20 was synthesized for electrical conductivity measurements. The green body was prepared via uniaxial dry pressing of BCNZ20 powder and subsequently sintered at 1200 °C. Archimedes’ drainage method confirmed a relative density exceeding 97%, sufficient for reliable transport property evaluation. Using the four-probe technique and a Keithley-2700 multimeter, the electrical conductivity was measured at 600, 550, and 500 °C and benchmarked against SOA BaCoO_3_-derived materials (Fig. S35).

(2) Oxygen transport properties: Electrical conductivity relaxation (ECR) measurements were conducted to extract the chemical surface exchange coefficient (*k*_Chem_) and chemical bulk diffusion coefficient (*D*_Chem_) for BCNZ20. By alternating the atmosphere between air and pure oxygen and monitoring changes in conductivity, the ORR/OER processes were simulated (Fig. S36a and b). A custom MATLAB script was used to fit the data and extract *k*_Chem_ and *D*_Chem_ across the temperature range 500-650 °C (Fig. S36c). Both metrics were compared with representative SOA oxygen electrode materials (Fig. S36d and e), highlighting BCNZ20’s favorable oxygen transport kinetics.

(3) Chemical Compatibility: The chemical compatibility of BCNZ20 with three electrolytes (SDC, BZCYYb1711, and BSCYb172) was examined. BCNZ20 powder was mechanically mixed with each electrolyte and calcinated at 950 °C for 2 hours. XRD analysis of the resulting mixtures (Fig. S37) showed that all peaks could be attributed to BCNZ20 or the corresponding electrolyte, confirming good chemical compatibility, critical for long-term interfacial stability in device operation.

(4) Thermal Expansion Coefficient: To assess thermal compatibility with electrolyte materials, TEC measurements were performed using bar-shaped BCNZ20 specimens. It exhibited a TEC of 16.9 E^-6^ K^-1^ below 450 °C, and 19.8 E^-6^ K^-1^ above 450 °C (Fig. S38a). These findings were validated by in-situ XRD-derived lattice parameters through Rietveld refinement, yielding TEC values of 14.9 E^-6^ K^-1^ below 450 °C and 19.2 E^-6^ K^-1^ above 450 °C, respectively (Fig. S38b). The inflection point near 450 °C is attributed to a spin-state transition of cobalt ions. Compared to benchmark ORR/OER materials, BCNZ20 displays a reduced TEC, supporting its enhanced thermo-mechanical stability (Fig. S38c).

(5) Steam-enhanced ORR/OER Kinetics: The effect of steam on ORR/OER performance was investigated through the EIS using BCNZ20/BZCYYb1711/BCNZ20 symmetrical cells under controlled humidified conditions. *R*_p_ values were measured from 500 to 700 °C in air containing 3 vol% H_2_O (Fig. S39), and compared with SOA ORR/OER electrocatalysts (Fig. S40). As the water vapor concentration was systematically increased from 3% to 10%, and then to 30%. With increasing humidity, EIS revealed a growing high-frequency arc – reflecting slowed oxygen dissociation – and a diminishing low-frequency arc, indicative of facilitated peroxide hydrogenation (Fig. S41).

Figure S1. Violin plots showing the distribution of (a) ${\bar{\boldsymbol{O}}}_{\boldsymbol{2}\boldsymbol{p}}$, (b) ${\bar{\boldsymbol{M}}}_{\boldsymbol{3}\boldsymbol{d}}$, (c) *E*_V_, (d) δ, and (e) *H_dp_* values across all 18 branches of perovskite candidates, with median and undoped pristine values highlighted for each branch.

Figure S2. Parity plots for (a) ${\bar{\boldsymbol{O}}}_{\boldsymbol{2}\boldsymbol{p}}$, (b) ${\bar{\boldsymbol{M}}}_{\boldsymbol{3}\boldsymbol{d}}$, (c) *E*_V_, (d) δ, and (e) *H_dp_*, predicted using the FSML model.

Figure S3. UMAP projection of the high-dimensional input feature space for 6,066 training compositions and 2,022 LaMO_3_-derived compositions.

Figure S4. Learning curve of the SSAL model in predicting *R*_p_.

Figure S5. Parity plots of *E*_a_ predictions generated by the SSAL model.

Figure S6. Cumulative error distribution for *E*_a_ predictions by the SSAL model: 168 features refer to the baseline model trained solely on 168 physicochemical attributes; 168 + GT includes both the 168 physicochemical features and ground-truth descriptors obtained from DFT calculations; 168 + FSML incorporates the 168 physicochemical features along with the FSML-predicted descriptors.

Figure S7. Parity plots for *R*_p_ of SOA ORR/OER electrocatalysts predicted using the SSAL model.

Figure S8. SHAP feature importance plot and beeswarm plots for *G*_hull_ using the FSML model. Feature names with an underscore indicate the corresponding combination type ($\mathcal{C}$).

Figure S9. (a-c) Distribution of *G*_hull_ as a function of electron affinity (feature $\mathcal{C}_{\boldsymbol{6}}$) across all 18 compositional branches of perovskite candidates. (d) Using BaCoO_3_-derived candidates as an example, the effect of each doping route on *G*_hull_ of pristine BaCoO_3_ is illustrated. The red line indicates the direction of increasing doping fraction.

Figure S10. SHAP feature importance and beeswarm plots for *G*_hull_ of BaCoO3-derived candidates using the FSML model. Feature names with an underscore indicate the corresponding combination type ($\mathcal{C}$).

Figure S11. SHAP feature importance and beeswarm plots for *R*_p_ of 568 candidates using the SSAL model. Feature names with an underscore indicate the corresponding combination type ($\mathcal{C}$).

Figure S12. Trends in two surface superoxide species properties – ICOHP, and delocalized electrons density – as a function of bulk *H_dp_* values for representative BaCoO_3_-derived compositions.

Figure S13. Schematically representation of the discovered structure-descriptor-performance pathway, connecting crystal structure, bulk *H_dp_*, surface ICOHP, electron delocalization, and *G*_ad, OO*_.

Figure S14. Partial-dependence plots (solid lines) and ICE curves (thin lines) showing the predicted *R*_p_ as a function of (a) *H_dp_* and (b) DensificationIndex.

Figure S15. Experimental *R*_p_ at 600 °C as a function of *H_dp_* and DensificationIndex ($\mathcal{C}_{\boldsymbol{2}}$) for across SOA electrocatalysts and all experimentally synthesized candidates in Stage Ⅱ.

Figure S16. SHAP feature importance plot and beeswarm plots for ${\bar{\boldsymbol{O}}}_{\boldsymbol{2}\boldsymbol{p}}$, ${\bar{\boldsymbol{M}}}_{\boldsymbol{3}\boldsymbol{d}}$, *E*_V_, δ, and *H_dp_*, using the FSML model. Feature names with an underscore indicate the corresponding combination type ($\mathcal{C}$).

Figure S17. Two-dimensional evaluation of Nb-doped BaCoO_3_, Zr-doped BaCoO_3_, and Nb, Zr co-doped BaCoO_3_ in terms of *H_dp_* and DensificationIndex ($\mathcal{C}_{\boldsymbol{2}}$). Arrows indicate the direction of increasing doping content.

Figure S18. BET analysis results - adsorption and desorption isotherms of BCNZ20.

**Figure S19.** Summary of EIS results for SDC symmetrical cells with Nb- and Zr-co-doped BaCoO_3_ across 700 to 500 °C, along with activation energies derived from Arrhenius analysis. (**a-c**) correspond to BaCo_0.9_Nb_0.05_Zr_0.05_O_3-δ_ (BCNZ10), (**d-f**) to BaCo_0.8_Nb_0.1_Zr_0.1_O_3-δ_ (BCNZ20), and (**g-i**) to BaCo_0.7_Nb_0.15_Zr_0.15_O_3-δ_ (BCNZ30). Specifically, BCNZ20 exhibits *R*_p_ values of 0.023, 0.038, 0.071, 0.157, and 0.353 Ω cm^2^ at 700, 650, 600, 550, 500 °C, respectively, outperforming both BCNZ10 and BCNZ30. Arrhenius analysis yields apparent activation energies of 0.92, 0.90, and 0.89 eV for BCNZ10, BCNZ20, and BCNZ30, respectively.

Figure S20. Comparative analysis of *R*_p_ for SDC symmetrical cells at 700-500 °C, highlighting SOA oxygen electrodes: La_0.6_Sr_0.4_Co_0.2_Fe_0.8_O_3-δ_ (LSCF)^1^, Ba_0.5_Sr_0.5_Co_0.8_Fe_0.2_O_3-δ_ (BSCF)^2^, Sm_0.5_Sr_0.5_CoO_3-δ_ (SSC)^3^, BaCo_0.4_Fe_0.4_Zr_0.1_Y_0.1_O_3-δ_ (BCFZY)^4^, PrBa_0.5_Sr_0.5_Co_1.5_Fe_0.5_O_5+δ_ (PBSCF)^5^, SrCo_0.8125_Sb_0.1875_O_3-δ_ (SCS)^18^, and PrBa_0.8_Ca_0.2_Co_2_O_5+δ_ (PBCC)^19^.

Figure S21. X-ray diffraction (XRD) patterns of BCNZ20.

Figure S22. SAED pattern of BCNZ20 powder, captured along the [111] zone axis, aligned parallel to the electron beam.

Figure S23. SAED pattern of BCNZ20 powder, captured along the [110] zone axis, aligned parallel to the electron beam.

Figure S24. SAED pattern of BCNZ20 powder, captured along the [001] zone axis, aligned parallel to the electron beam.

Figure S25. Line-scan analysis of BCNZ20 based on the HAADF-STEM image and corresponding EDS elemental maps.

Figure S26. TEM image of BCNZ20 powder with corresponding EDS mapping and spectra.

Figure S27. Contour plots of in-situ XRD patterns for BCNZ20 in (a) air and (b) wet air, with lattice parameters provided at different temperature.

Figure S28. Detailed energy profiles and variations in Bader charges for oxygen atoms $\boldsymbol{O}_{\mathbf{Ⅰ}}$ and $\boldsymbol{O}_{\mathbf{Ⅱ}}$ during ORR on BO_2_-terminated (001) BCNZ20 surface.

Figure S29. Conventional ORR/OER rate-determining steps identification (a) EIS results for BCNZ20/SDC/BCNZ20 symmetrical cells at 600°C under various oxygen partial pressure. (b) DRT analysis of the EIS results presented in (a). (c) The results of linear fitting for the logarithm of both high-frequency and low-frequency *R*_p_, plotted as functions of the logarithm of oxygen partial pressure.

Figure S30. Top and side views of the configurations during ORR on the BO_2_-terminated (001) BCNZ20 surface.

Figure S31. Detailed energy profiles and variations in Bader charges for oxygen atoms $\boldsymbol{O}_{\mathbf{Ⅰ}}$ and $\boldsymbol{O}_{\mathbf{Ⅱ}}$ during PI-ORR on BO_2_-terminated (001) BCNZ20 surface.

Figure S32. Top and side views of the configurations during PI-ORR on the BO_2_-terminated (001) BCNZ20 surface.

Figure S33. XPS analysis of surface O 1s spectrums for (a) as-prepared BCNZ20, and (b-d) BCNZ20, BCFN, and PBC powders subjected to a 24-hour pretreatment in wet air immediately prior to measurement. (e) Direct comparison of the relative contributions of surface oxygen species across all samples.

Figure S34. Programmed heating thermogravimetric analysis performed on BCNZ20. A mass change of 0.04wt.% is observed at 600 °C upon hydration induced by 3% H_2_O.

Figure S35. Comparison of the electrical conductivity between BCNZ20 and the SOA materials.

Figure S36. Normalized conductivity curves for BCNZ20 measured between 500 °C and 600 °C during ECR experiments under (a) ORR and (b) OER. (c) Two metrics, *k*_Chem_, and *D*_Chem_, are assessed under conditions of elevated oxygen partial pressure (ORR) and decreased oxygen partial pressure (OER). Comparison of the (d) *k*_Chem_, and (e) *D*_Chem_ between BCNZ20 and the SOA materials.

Figure S37. XRD patterns demonstrating the chemical compatibility between BCNZ20 and three electrolyte materials, SDC, BZCYYb1711, and BSCYb172.

Figure S38. Lattice expansion curves for BCNZ20, derived from in-situ XRD measurements (a), and thermal expansion curves (b) for BCNZ20, derived from strip samples evaluation. (c) Comparison of the TEC between BCNZ20 and the SOA materials.

Figure S39. EIS results for BZCYYb1711 symmetrical cells with BCNZ20 as the oxygen electrode across temperature from 700°C to 500°C.

Figure S40. Comparative analysis of *R*_p_ for BZCYYb1711 symmetrical cells at 700-500 °C, highlighting SOA oxygen electrodes: Ba_0.5_Sr_0.5_(Co_0.8_Fe_0.2_)_0.95_P_0.05_O_3-δ_ (BSCFP) ^20^, Pr_0.1_Ce_0.9_O_2_-PrBaCo_2_O_5+δ_ (PCO-PBC) ^21^, BaCo_0.4_Fe_0.4_Zr_0.1_Y_0.1_O_3-δ_ (BCFZY) ^22^, PrBa_0.8_Ca_0.2_Co_2_O_5+δ_ (PBCC) ^19^, PrBaCo_2_O_5+δ_ -BaCoO_3-δ_ (PBC-BCO) ^23^, Ba_0.9_Co_0.7_Fe_0.2_Nb_0.1_O_3-δ_ (BCFN) ^24^, Pr_0.2_Ba_0.2_Sr_0.2_La_0.2_Ca_0.2_CoO_3-δ_ (PBSLCC) ^25^, Ba_2_Co_1.5_Mo_0.25_Nb_0.25_O_6-δ_ (BCMN) ^26^, Gd_0.3_Ca_2.7_Co_3.82_Cu_0.18_O_9-δ_ (GCCC) ^27^.

Figure S41. (a) EIS results for BCNZ20/BZCYYb1711/BCNZ20 symmetrical cells at 600 °C under various water vapor pressures using air as the carrier gas. (b) DRT analysis of the EIS results presented in (a), focusing on the identification of different elementary steps.

Figure S42. EIS results for single cells at OCV under 600-400 °C with oxygen electrode fed by (a-c) air and (c-e) oxygen.

Figure S43. Representative *I-V-P* curves measured at 600-400 °C in fuel cell mode with oxygen electrode fed by air.

Figure S44. Reproducibility evaluation of the fabricated P-SOCs single cells with the same configuration. (a) Representative *I-V-P* curves and (b) corresponding EIS results under OCV conditions at 600 °C in fuel cell mode with oxygen electrodes fed by air, obtained from three independent trails.

Figure S45. Comparative analysis of current densities at 1.3 V for P-SOCs at 600–400 °C, highlighting SOA oxygen electrodes: PrBaCo_1.9_Hf_0.1_O_5+δ_ (PBCHf)^14^, Ba_0.1_Pr_0.9_Hf_0.1_Y_0.1_Co_0.8_O_3-δ_ (BPHYC)^28^, PrBa_0.8_Ca_0.2_Co_2_O_5+δ_ (PBCC)^29^, BaCo_0.4_Fe_0.4_Zr_0.1_Y_0.1_O_3-δ_ (BCFZY)^4^, PrBa_0.5_Sr_0.5_Co_1.5_Fe_0.5_O_5+δ_ (PBSCF)^5^, Ba_0.9_Co_0.7_Fe_0.2_Nb_0.1_O_3-δ_ (BCFN)^24^.

Figure S46. Electrochemical performance of PCEC single cells using BaCo_0.75_Hf_0.125_Zr_0.125_O_3_ (No. 1 in Fig. 4c) as the oxygen electrode. (a, b) Representative *I-V-P* curves measured in fuel cell mode at 600-500 °C with oxygen electrode fed by oxygen (a) and air (b). (c) Representative *I-V* curves measured in electrolysis cell mode at 600-500 °C. (d, e) EIS results for single cells at OCV under 600-500 °C with oxygen electrode fed by oxygen (d) and air (e).

Figure S47. Electrochemical performance of PCEC single cells using BaCo_0.875_Sc_0.0625_Y_0.0625_O_3_ (No. 5 in Fig. 4c) as the oxygen electrode. (a, b) Representative *I-V-P* curves measured in fuel cell mode at 600-500 °C with oxygen electrode fed by oxygen (a) and air (b). (c) Representative *I-V* curves measured in electrolysis cell mode at 600-500 °C. (d, e) EIS results for single cells at OCV under 600-500 °C with oxygen electrode fed by oxygen (d) and air (e).

Figure S48. Electrochemical performance of PCEC single cells using BaCo_0.875_Nb_0.0625_Sc_0.0625_O_3_ (No. 16 in Fig. 4c) as the oxygen electrode. (a, b) Representative *I-V-P* curves measured in fuel cell mode at 600-500 °C with oxygen electrode fed by oxygen (a) and air (b). (c) Representative *I-V* curves measured in electrolysis cell mode at 600-500 °C. (d, e) EIS results for single cells at OCV under 600-500 °C with oxygen electrode fed by oxygen (d) and air (e).

Figure S49. Electrochemical performance of PCEC single cells using BaCo_0.75_Sc_0.125_Dy_0.125_O_3_ (No. 23 in Fig. 4c) as the oxygen electrode. (a, b) Representative *I-V-P* curves measured in fuel cell mode at 600-500 °C with oxygen electrode fed by oxygen (a) and air (b). (c) Representative *I-V* curves measured in electrolysis cell mode at 600-500 °C. (d, e) EIS results for single cells at OCV under 600-500 °C with oxygen electrode fed by oxygen (d) and air (e).

Figure S50. Long-term reversible operation durability of BCNZ20 single cell: The cell voltage variations over time when the operating mode is alternated between fuel cell and electrolysis every 2 hours, with 3% water vapor pressure present in the oxygen electrode.

Figure S51. SEM images of the BCNZ20 oxygen electrode showing (a) the pristine state prior to electrochemical testing, (b) after 250 h of operation in FC mode, and (c) after 500 h of operation in EC mode. (d) XRD patterns of the BCNZ20 oxygen electrode in the pristine, post-FC, and post-EC states.

**Figure S52.** (**a**) Electrochemical impedance spectroscopy (EIS) plots and (**b**) representative I-V-P curves for a BCNZ20/SDC/YSZ/YSZ-NiO single cell measured at 700-600 °C. (**c**) Long-term galvanostatic tests of the same single cell at 600 °C under fuel cell mode at a current density of 0.3 A cm^-2^. The cell exhibits ohmic resistances of 0.155, 0.226, and 0.354 Ω cm^2^ and polarization resistances of 0.128, 0.220, and 0.363 Ω cm^2^ at 700, 650, and 600 °C, respectively. Under operating conditions of wet H_2_ containing 3 vol% H_2_O at the fuel electrode and ambient air at the oxygen electrode, the cell delivers peak power densities of 1.60, 1.14, and 0.68 W cm^-2^ at 700, 650, 600 °C, respectively. Furthermore, the cell demonstrates excellent durability, with negligible performance degradation during a 400-hour constant-current fuel-cell test.

Table S1. Summary of optimized XGBoost hyperparameters for predicting different descriptors in both learning stages

| **Hyperparameters** | **Descriptors** | | | | | | | |
| --- | --- | --- | --- | --- | --- | --- | --- | --- |
|  | *G*_hull_ | *p*-band center | *d-*band center | *d-p* hybridization | *E*_V_ | δ | *R*_p_ | *E*_a_ |
| Max_depth | 6 | 6 | 8 | 6 | 4 | 4 | 6 | 6 |
| Learning_rate | 0.1 | 0.1 | 0.1 | 0.1 | 0.1 | 0.1 | 0.1 | 0.1 |
| N_estimators | 200 | 200 | 200 | 200 | 200 | 200 | 200 | 200 |
| Max_leaves | 0 | 0 | 0 | 0 | 0 | 0 | 0 | 0 |
| Min_child_weight | 10 | 5 | 5 | 10 | 5 | 10 | 20 | 20 |

Table S2. Complete list of 168 input features used in the FSML model. Features are organized by attribute category (A1: Atomic, A2: Bonding, A3: Structural). Each base property in A1 and A2 is expanded through twelve linear combinations ($\mathcal{C}_{\boldsymbol{1}}\boldsymbol{\sim}\mathcal{C}_{\boldsymbol{12}}$, defined in Supplementary Note S2). Source: C = composition-derived; DFT = DFT-derived; ML = ML-predicted.

| No. | Feature name | Base property | Definition | Unit | Source | Category |
| --- | --- | --- | --- | --- | --- | --- |
| 1 | Elemental Type for A-site major | Elemental type (Z) | One-hot of A-site major | - | C | A1 |
| 2 | Elemental Type for A-site dopant | Elemental type (Z) | One-hot of A-site dopant | - | C | A1 |
| 3 | Elemental Type for B-site major | Elemental type (Z) | One-hot of B-site major | - | C | A1 |
| 4 | Elemental Type for B-site dopant | Elemental type (Z) | One-hot of B-site dopant | - | C | A1 |
| 5 | Stoichiometry for A-site major | Stoichiometry (N) | One-hot of A-site major | - | C | A1 |
| 6 | Stoichiometry for A-site dopant | Stoichiometry (N) | One-hot of A-site dopant | - | C | A1 |
| 7 | Stoichiometry for B-site major | Stoichiometry (N) | One-hot of B-site major | - | C | A1 |
| 8 | Stoichiometry for B-site dopant | Stoichiometry (N) | One-hot of B-site dopant | - | C | A1 |
| 9-20 | ElectronAffinity $\mathcal{C}_{1}\sim\mathcal{C}_{12}$ | Electron affinity | Electron affinity with 12 distinct linear combinations | eV | C | A1 |
| 21-32 | Oxidation state $\mathcal{C}_{1}\sim\mathcal{C}_{12}$ | Oxidation state | Oxidation state with 12 distinct linear combinations | - | C | A1 |
| 33-44 | Melting point $\mathcal{C}_{1}\sim\mathcal{C}_{12}$ | Melting point | Melting point with 12 distinct linear combinations | K | C | A1 |
| 45-56 | Boiling point $\mathcal{C}_{1}\sim\mathcal{C}_{12}$ | Boiling point | Boiling point with 12 distinct linear combinations | K | C | A1 |
| 57-68 | Atomic mass $\mathcal{C}_{1}\sim\mathcal{C}_{12}$ | Atomic mass | Atomic mass with 12 distinct linear combinations | g/mol | C | A1 |
| 69-80 | Elemental density $\mathcal{C}_{1}\sim\mathcal{C}_{12}$ | Elemental density | Elemental density with 12 distinct linear combinations | g/cm³ | C | A1 |
| 81-92 | Ionization energy $\mathcal{C}_{1}\sim\mathcal{C}_{12}$ | Ionization energy | Ionization energy with 12 distinct linear combinations | kJ/mol | C | A1 |
| 93-104 | Electronegativity $\mathcal{C}_{1}\sim\mathcal{C}_{12}$ | Electronegativity | Electronegativity with 12 distinct linear combinations | - | C | A1 |
| 105-116 | Chemical potential of the oxide $\mathcal{C}_{1}\sim\mathcal{C}_{12}$ | Chemical potential of the oxide | Chemical potential of the oxide with 12 distinct linear combinations | eV | C | A1 |
| 117-128 | Radius $\mathcal{C}_{1}\sim\mathcal{C}_{12}$ | Shannon radius | Shannon radius with 12 distinct linear combinations | Å | C | A2 |
| 129-140 | DensificationIndex $\mathcal{C}_{1}\sim\mathcal{C}_{12}$ | Melting points of the precursors | Melting points of the precursors with 12 distinct linear combinations | K | C | A2 |
| 141-152 | Bond length $\mathcal{C}_{1}\sim\mathcal{C}_{12}$ | DFT calculated bond length | DFT calculated bond length with 12 distinct linear combinations | Å | DFT | A2 |
| 153-164 | COHP $\mathcal{C}_{1}\sim\mathcal{C}_{12}$ | Crystal Orbital Hamilton Population | Crystal Orbital Hamilton Population with 12 distinct linear combinations | eV | DFT | A2 |
| 165 | Tolerance factor | Tolerance Factor | Goldschmidt tolerance factor | - | C | A3 |
| 166 | Modified tolerance factor | Modified tolerance factor | Modified tolerance factor | - | C | A3 |
| 167 | Octahedral factor | Octahedral factor | Octahedral factor | - | C | A3 |
| 168 | δ | Oxygen non-stoichiometry | ML-predicted from Li et al. dataset | - | ML | A3 |

Table S3. Chemical composition of the training and test sets under the 4:1 split for both FSML (Stage I) and SSAL (Stage II) models. Note: For Stage I, a “dopant route” is defined as a unique combination of parent lattice and dopant identity (e.g., BaCo-Nb). For Stage II, entries correspond to composition-temperature pairs; thus, the same composition measured at different temperatures may appear in both training and test sets.

| Category | Stage I Train | Stage I Test | Shared | Stage II Train | Stage II Test | Shared |
| --- | --- | --- | --- | --- | --- | --- |
| Compositions | 4,853 | 1,213 | 0 | 348 | 87 | 0 |
| A-site elements | 3 | 3 | 3 | 3 | 3 | 3 |
| Parent Lattices | 18 | 18 | 18 | 6 | 6 | 6 |
| B-site Branches | 6 | 6 | 6 | 2 | 2 | 2 |
| Dopant Elements | 42 | 42 | 42 | 17 | 14 | 14 |
| Dopant Routes | 337 | 337 | 337 | 38 | 24 | 21 |
| Dopant Fractions | 9 | 9 | 9 | 5 | 4 | 4 |

Table S4. Pseudopotentials and *U*_eff_ values used in the high-throughput computations

| **Element** | **Pseudopotential** | ***U*_eff_ (eV)** |
| --- | --- | --- |
| Ba | Ba_sv 06Sep2000 | 0 |
| Sr | Sr_sv 07Sep2000 | 0 |
| Pr | Pr_3 07Sep2000 | 0 |
| Cr | Cr_pv 02Aug2007 | 3.7 |
| Mn | Mn_pv 02Aug2007 | 3.9 |
| Fe | Fe_pv 02Aug2007 | 5.3 |
| Co | Co 02Aug2007 | 3.32 |
| Ni | Ni_pv 06Sep2000 | 6.2 |
| Cu | Cu_pv 06Sep2000 | 0 |
| Al | Al 04Jan2001 | 0 |
| As | As 22Sep2009 | 0 |
| Bi | Bi 08Apr2002 | 0 |
| Ca | Ca_sv 06Sep2000 | 0 |
| Ce | Ce 23Dec2003 | 0 |
| Dy | Dy_3 06Sep2000 | 0 |
| Er | Er_3 06Sep2000 | 0 |
| Eu | Eu_3 20Oct2008 | 0 |
| Ga | Ga_d 06Jul2010 | 0 |
| Gd | Gd_3 06Sep2000 | 0 |
| Ge | Ge_d 03Jul2007 | 0 |
| Hf | Hf_pv 06Sep2000 | 0 |
| Ho | Ho_3 06Sep2000 | 0 |
| In | In 08Apr2002 | 0 |
| La | La 06Sep2000 | 0 |
| Lu | Lu_3 06Sep2000 | 0 |
| Mg | Mg_pv 13Apr2007 | 0 |
| Mo | Mo_pv 04Feb2005 | 4.38 |
| Nb | Nb_pv 08Apr2002 | 0 |
| Nd | Nd_3 06Sep2000 | 0 |
| Pb | Pb 08Apr2002 | 0 |
| Sb | Sb 06Sep2000 | 0 |
| Sc | Sc_sv 07Sep2000 | 0 |
| Sm | Sm_3 07Sep2000 | 0 |
| Sn | Sn_d 06Sep2000 | 0 |
| Ta | Ta_pv 07Sep2000 | 0 |
| Tb | Tb_3 06Sep2000 | 0 |
| Te | Te 08Apr2002 | 0 |
| Ti | Ti_pv 07Sep2000 | 0 |
| Tm | Tm_3 20Jan2003 | 0 |
| V | V_pv 07Sep2000 | 3.25 |
| W | W 08Apr2002 | 0 |
| Y | Y_sv 25May2007 | 0 |
| Yb | Yb_2 06Sep2000 | 0 |
| Zn | Zn 06Sep2000 | 0 |
| Zr | Zr_sv 04Jan2005 | 0 |
| O | O 08Apr2002 | 0 |

Table S5. Root mean squared error (RMSE) for various algorithms used in the FSML model for predicting computational descriptors on the test set

| **Algorithm** | **RMSE (RMSE with normalized data) on the test set** | | | | | |
| --- | --- | --- | --- | --- | --- | --- |
|  | *G*_hull_ (eV/atom) | *p*-band center (eV) | *d-*band center (eV) | *d-p* hybridization | *E*_V_ (eV) | δ |
| LASSO | 0.17321 (0.22884) | 0.20579 (0.16667) | 0.28679 (0.22847) | 0.43176 (0.14018) | 0.69216 (0.17187) | 0.14903  (0.17742) |
| SVR | 0.17499 (0.23119) | 0.20426 (0.16544) | 0.29198 (0.23262) | 0.43927 (0.14262) | 0.69075 (0.17152) | 0.149025  (0.177410) |
| RF | 0.17187 (0.22707) | 0.31659 (0.25642) | 0.26892 (0.21424) | 0.40780 (0.13240) | 0.77827 (0.19326) | 0.227354  (0.270659) |
| ANN | 0.02565 (0.03393) | 0.04594 (0.03715) | 0.09471 (0.07498) | 0.14349 (0.04658) | 0.28190 (0.07000) | 0.109485  (0.130339) |
| XGBoost | 0.02087 (0.02757) | 0.04604 (0.03728) | 0.08143 (0.06489) | 0.12243 (0.03975) | 0.27970 (0.06943) | 0.109239  (0.130046) |
| XGBoost on leave-one-route-out task | 0.02517  (0.03325) | 0.08261  (0.06689) | 0.08728  (0.06955) | 0.26113  (0.08478) | 0.41822  (0.10381) | 0.11025  (0.13125) |
| XGBoost on out-of-distribution task | 0.03439 (0.04765) | 0.19723 (0.091488) | 0.09220 (0.09793) | 0.61945 (0.20112) | 0.72398 (0.13315) | 0.112376  (0.133781) |

Table S6. Comparison of RMSE and mean absolute error (MAE) in predicting *G*_hull_ and Gibbs formation energy (*G*_form_) between the FSML model and several SOA models

| **Model** | ***G*_hull_ (RMSE)** | ***G*_hull_ (MAE)** | ***G*_form_ (RMSE)** | ***G*_form_ (MAE)** | **Ref** |
| --- | --- | --- | --- | --- | --- |
| FSML (XGBoost) | 20.87 | 11.63 | 20.65 | 11.39 | This work |
| Ye’s Model |  |  |  | 20~34 | ^30^ |
| Schmidt’s Model |  | 121 |  |  | ^31^ |
| Li’s Model | 29.4 | 16.0 |  |  | ^32^ |
| Chen’s Model |  |  |  | 28 | ^33^ |
| Cheng’s Model |  |  |  | 16.07~31.66 | ^34^ |
| Jonathan’s Model |  | 41 |  |  | ^35^ |

Unit: meV/atom

Table S7. Convergence metrics of the SSAL process across seven acquisition rounds. All RMSE and *R*^2^ values are reported in log(*R*_p_) space. Spearman ρ: rank correlation of predicted *R*_p_ for all candidates in the screening pool between the current and previous round. Top-10 overlap: number of shared candidates in the top-10 rankings between consecutive rounds.

| Round | Ntotal | RMSE | *R*^2^ | Spearman ρ | Top-10 overlap |
| --- | --- | --- | --- | --- | --- |
| 1 | 240 | 0.077 | 0.947 | - | - |
| 2 | 288 | 0.053 | 0.958 | 0.870 | 7/10 |
| 3 | 336 | 0.032 | 0.959 | 0.887 | 8/10 |
| 4 | 384 | 0.024 | 0.962 | 0.950 | 8/10 |
| 5 | 432 | 0.0213 | 0.963 | 0.965 | 9/10 |
| 6 | 480 | 0.0204 | 0.972 | 0.967 | 9/10 |
| 7 | 568 | 0.02 | 0.980 | 0.971 | 10/10 |

Table S8. Complete candidate-selection log of the SSAL process. All 145 unique compositions are listed with their acquisition round, experimentally measured *R*_p_ (Ω cm^2^) at 700, 650, and 600 °C, and activation energy *E*_a_ (eV). Within each round, compositions are sorted by *R*_p_ at 600 °C. “–” indicates the measurement was not available. The total dataset comprises 568 entries (435 *R*_p_ measurements at three temperatures + 133 *E*_a_ values)

| **Round** | **Composition** | **x** | ***R*_p_ at 700°C** | ***R*_p_ at 650°C** | ***R*_p_ at 600°C** | ***E*_a_ (eV)** |
| --- | --- | --- | --- | --- | --- | --- |
| 0 | BaCo-In | 0.125 | 0.025 | 0.044 | 0.081 | 0.844 |
| 0 | SrCo-Sb | 0.0625 | 0.033 | 0.055 | 0.104 | 0.831 |
| 0 | BaCo-Nb | 0.0625 | 0.034 | 0.059 | 0.114 | 1.162 |
| 0 | BaCo-Zr | 0.0625 | 0.045 | 0.114 | 0.121 | 1.429 |
| 0 | BaCo-Sc | 0.0625 | 0.027 | 0.050 | 0.127 | 0.805 |
| 0 | BaCo-Sb | 0.0625 | 0.081 | 0.076 | 0.129 | 1.148 |
| 0 | SrCo-Hf | 0.0625 | 0.037 | 0.068 | 0.138 | 0.971 |
| 0 | BaCo-W | 0.0625 | 0.045 | 0.076 | 0.140 | 1.181 |
| 0 | BaCo-Sn | 0.0625 | 0.052 | 0.082 | 0.145 | 1.268 |
| 0 | BaCo-In | 0.0625 | 0.052 | 0.082 | 0.146 | 1.125 |
| 0 | BaCo-Y | 0.125 | 0.046 | 0.090 | 0.155 | 0.886 |
| 0 | BaCo-Zn | 0.0625 | 0.050 | 0.086 | 0.162 | 0.866 |
| 0 | BaCo-Nb | 0.125 | 0.050 | 0.104 | 0.165 | 0.861 |
| 0 | BaCo-Ta | 0.0625 | 0.050 | 0.157 | 0.170 | 1.153 |
| 0 | BaCo-Ce | 0.0625 | 0.049 | 0.091 | 0.173 | 1.237 |
| 0 | BaCo-Mo | 0.0625 | 0.056 | 0.166 | 0.183 | 0.796 |
| 0 | BaCo-In | 0.25 | 0.033 | 0.061 | 0.203 | 1.127 |
| 0 | BaCo-Ga | 0.0625 | 0.068 | 0.121 | 0.226 | 0.875 |
| 0 | BaFe-Hf | 0.0625 | 0.070 | 0.112 | 0.229 | 1.308 |
| 0 | SrCo-Zr | 0.0625 | 0.064 | 0.117 | 0.235 | 0.946 |
| 0 | BaFe-Sc | 0.0625 | 0.063 | 0.110 | 0.235 | 0.780 |
| 0 | BaCo-Y | 0.0625 | 0.117 | 0.141 | 0.236 | 1.209 |
| 0 | BaCo-Zr | 0.125 | 0.100 | 0.183 | 0.237 | 0.629 |
| 0 | SrCo-Sc | 0.0625 | 0.080 | 0.125 | 0.249 | 0.837 |
| 0 | BaFe-Nb | 0.0625 | 0.075 | 0.141 | 0.253 | 1.250 |
| 0 | BaFe-Sb | 0.0625 | 0.108 | 0.373 | 0.263 | 1.262 |
| 0 | BaCo-Hf | 0.0625 | 0.029 | 0.054 | 0.267 | 1.222 |
| 0 | BaCo-Bi | 0.0625 | 0.078 | 0.139 | 0.270 | 0.906 |
| 0 | BaCo-Hf | 0.125 | 0.075 | 0.194 | 0.290 | 0.983 |
| 0 | BaFe-W | 0.0625 | 0.105 | 0.173 | 0.301 | 1.065 |
| 0 | BaFe-In | 0.0625 | 0.224 | 0.173 | 0.315 | 1.160 |
| 0 | BaFe-Y | 0.125 | 0.097 | 0.184 | 0.316 | 0.864 |
| 0 | SrFe-Hf | 0.0625 | 0.075 | 0.152 | 0.322 | 1.072 |
| 0 | BaFe-Sn | 0.0625 | 0.235 | 0.168 | 0.325 | 1.238 |
| 0 | BaCo-Yb | 0.125 | 0.068 | 0.131 | 0.345 | 1.197 |
| 0 | BaFe-Nb | 0.125 | 0.105 | 0.248 | 0.360 | 0.896 |
| 0 | BaFe-Ce | 0.0625 | 0.105 | 0.218 | 0.364 | 1.311 |
| 0 | BaCo-Ta | 0.125 | 0.077 | 0.157 | 0.370 | 1.147 |
| 0 | BaFe-Zn | 0.0625 | 1.813 | 0.185 | 0.390 | 0.877 |
| 0 | BaCo-Sb | 0.125 | 0.095 | 0.183 | 0.429 | 1.102 |
| 0 | BaFe-Ga | 0.0625 | 0.141 | 0.250 | 0.464 | 0.871 |
| 0 | BaFe-Yb | 0.0625 | 0.154 | 0.288 | 0.476 | 1.332 |
| 0 | BaCo-W | 0.125 | 0.116 | 0.257 | 0.495 | 1.059 |
| 0 | SrFe-Zr | 0.0625 | 0.134 | 0.280 | 0.498 | 0.957 |
| 0 | BaFe-Y | 0.0625 | 0.163 | 0.502 | 0.537 | 1.169 |
| 0 | BaCo-Ce | 0.125 | 0.104 | 0.232 | 0.554 | 1.225 |
| 0 | BaFe-Zr | 0.125 | 0.239 | 0.433 | 0.565 | 0.626 |
| 0 | SrFe-Sc | 0.0625 | 0.188 | 0.287 | 0.595 | 0.845 |
| 0 | BaFe-Mo | 0.0625 | 0.116 | 0.223 | 0.612 | 0.744 |
| 0 | BaFe-Bi | 0.0625 | 0.164 | 0.284 | 0.633 | 0.991 |
| 0 | BaCo-Sn | 0.125 | 0.132 | 0.273 | 0.676 | 1.197 |
| 0 | BaFe-Hf | 0.125 | 0.171 | 0.469 | 0.699 | 1.020 |
| 0 | BaFe-Yb | 0.125 | 0.149 | 0.313 | 0.699 | 1.130 |
| 0 | BaFe-Ta | 0.0625 | 0.108 | 0.178 | 0.873 | 1.268 |
| 0 | BaFe-Ta | 0.125 | 0.171 | 0.343 | 0.902 | 1.220 |
| 0 | BaFe-Sb | 0.125 | 0.217 | 0.437 | 0.914 | 1.052 |
| 0 | BaFe-Zr | 0.0625 | 0.111 | 0.155 | 0.925 | 1.515 |
| 0 | BaCo-Yb | 0.0625 | 0.070 | 0.119 | 0.951 | 1.247 |
| 0 | BaFe-W | 0.125 | 0.246 | 0.581 | 1.125 | 1.108 |
| 0 | BaFe-Ce | 0.125 | 0.249 | 0.542 | 1.134 | 1.107 |
| 0 | BaCo-Mo | 0.125 | 0.242 | 0.588 | 1.521 | 1.344 |
| 0 | BaFe-Sn | 0.125 | 0.296 | 0.651 | 1.532 | 1.204 |
| 0 | BaCo-Sc | 0.125 | 0.447 | 0.803 | 1.607 | 0.937 |
| 0 | BaFe-In | 0.125 | 0.512 | 1.075 | 2.839 | 1.256 |
| 0 | BaFe-Mo | 0.125 | 0.499 | 1.326 | 3.650 | 1.456 |
| 0 | BaFe-Sc | 0.125 | 0.928 | 1.792 | 3.719 | 1.017 |
| 0 | BaCo-Bi | 0.125 | 0.972 | -- | -- | -- |
| 0 | BaFe-Bi | 0.125 | 1.973 | -- | -- | -- |
| 1 | BaCo-Hf | 0.5 | 0.045 | 0.101 | 0.209 | 1.119 |
| 1 | SrFe-Sb | 0.0625 | 0.078 | 0.119 | 0.225 | 0.782 |
| 1 | BaCo-Hf | 0.25 | 0.068 | 0.137 | 0.269 | 1.005 |
| 1 | BaCo-In | 0.5 | 0.108 | 0.214 | 0.544 | 1.190 |
| 1 | BaCo-Ce | 0.5 | 0.284 | 0.547 | 0.881 | 0.825 |
| 1 | BaCo-Ce | 0.25 | 0.308 | 0.627 | 1.200 | 0.994 |
| 1 | BaCo-Mo | 0.25 | 0.358 | 0.849 | 2.399 | 1.393 |
| 2 | BaCo-Nb | 0.25 | 0.039 | 0.079 | 0.160 | 1.034 |
| 2 | BaCo-Sb | 0.5 | 0.065 | 0.124 | 0.276 | 1.062 |
| 2 | BaCo-Nb | 0.5 | 0.059 | 0.117 | 0.301 | 1.191 |
| 2 | BaCo-Sn | 0.25 | 0.068 | 0.146 | 0.362 | 1.222 |
| 2 | BaCo-Sc | 0.5 | 0.076 | 0.164 | 0.488 | 1.361 |
| 2 | BaCo-Sc | 0.25 | 0.180 | 0.438 | 1.251 | 1.422 |
| 2 | BaCo-Sb | 0.25 | 0.292 | 0.641 | 1.688 | 1.285 |
| 2 | BaCo-Mo | 0.5 | 0.559 | 1.284 | 3.243 | 1.288 |
| 3 | BaCo-Ta | 0.5 | 0.035 | 0.093 | 0.201 | 1.284 |
| 3 | BaCo-Ta | 0.25 | 0.053 | 0.105 | 0.238 | 1.098 |
| 3 | BaCo-Sn | 0.5 | 0.076 | 0.163 | 0.400 | 1.214 |
| 3 | BaCo-Yb | 0.5 | 0.072 | 0.205 | 0.608 | 1.565 |
| 3 | BaCo-Y | 0.5 | 0.123 | 0.338 | 1.019 | 1.546 |
| 3 | BaCo-Yb | 0.25 | 0.138 | 0.381 | 1.131 | 1.538 |
| 3 | BaCo-Y | 0.25 | 0.158 | 0.426 | 1.391 | 1.596 |
| 3 | BaCo-Zr | 0.25 | 0.313 | 0.726 | 2.224 | 1.437 |
| 4 | BaFe-Hf | 0.5 | 0.105 | 0.220 | 0.428 | 1.030 |
| 4 | BaFe-In | 0.25 | 0.089 | 0.192 | 0.432 | 1.154 |
| 4 | BaFe-Hf | 0.25 | 0.148 | 0.299 | 0.589 | 1.011 |
| 4 | BaCo-Zr | 0.5 | 0.158 | 0.361 | 0.730 | 1.118 |
| 4 | BaFe-In | 0.5 | 0.233 | 0.478 | 1.130 | 1.156 |
| 4 | BaFe-Ce | 0.5 | 0.674 | 1.177 | 2.130 | 0.842 |
| 4 | BaFe-Ce | 0.25 | 0.625 | 1.269 | 2.622 | 1.049 |
| 4 | BaFe-Mo | 0.25 | 0.848 | 1.971 | 5.567 | 1.379 |
| 5 | BaFe-Nb | 0.25 | 0.080 | 0.185 | 0.340 | 1.055 |
| 5 | BaFe-Sb | 0.5 | 0.156 | 0.250 | 0.568 | 0.948 |
| 5 | BaFe-Nb | 0.5 | 0.134 | 0.261 | 0.616 | 1.119 |
| 5 | BaFe-Sn | 0.25 | 0.162 | 0.318 | 0.774 | 1.146 |
| 5 | BaFe-Sc | 0.5 | 0.154 | 0.396 | 0.987 | 1.358 |
| 5 | BaFe-Sc | 0.25 | 0.383 | 0.990 | 2.858 | 1.472 |
| 5 | BaFe-Sb | 0.25 | 0.625 | 1.563 | 3.757 | 1.311 |
| 5 | BaFe-Mo | 0.5 | 1.270 | 2.715 | 6.851 | 1.235 |
| 6 | BaCo-Al | 0.0 | 0.039 | 0.068 | 0.329 | 1.193 |
| 6 | BaFe-Ta | 0.5 | 0.080 | 0.227 | 0.419 | 1.206 |
| 6 | BaFe-Ta | 0.25 | 0.112 | 0.251 | 0.489 | 1.078 |
| 6 | BaFe-Sn | 0.5 | 0.173 | 0.379 | 0.847 | 1.161 |
| 6 | BaFe-Yb | 0.5 | 0.153 | 0.420 | 1.370 | 1.606 |
| 6 | BaFe-Zr | 0.5 | 0.374 | 0.729 | 1.667 | 1.095 |
| 6 | BaFe-Y | 0.5 | 0.251 | 0.769 | 2.196 | 1.586 |
| 6 | BaFe-Yb | 0.25 | 0.301 | 0.856 | 2.357 | 1.504 |
| 6 | BaFe-Y | 0.25 | 0.359 | 0.881 | 3.356 | 1.641 |
| 6 | BaFe-Zr | 0.25 | 0.656 | 1.589 | 4.804 | 1.459 |

Table S9. Five-fold cross-validation RMSE for different configurations of the SSAL model in predicting experimental metrics (*R*_p_ and *E*_a_), compared with several SOA models

| **Model** | **RMSE of log (*R*_p_)** | **RMSE of *E*_a_ (eV)** | **Ref** |
| --- | --- | --- | --- |
| XGBoost (168 features) | 0.110 | 0.028 | This work |
| XGBoost (168 features + GT) | 0.015 | 0.016 | This work |
| XGBoost (168 features + FSML) | 0.020 | 0.019 | This work |
| XGBoost (168 features + FSML) | 0.021 @ 700 °C |  | This work |
| XGBoost (168 features + FSML) | 0.018 @ 650 °C |  | This work |
| XGBoost (168 features + FSML) | 0.020 @ 600 °C |  | This work |
| Zhai’s Model | 0.336 |  | ^36^ |
| Ryan’s Model | 0.590 |  | ^37^ |

Table S10. Three-dimensional evaluation of SOA catalysts in terms of cost effectiveness, thermodynamic stability, and electrocatalytic activity: La_0.6_Sr_0.4_Co_0.2_Fe_0.8_O_3-δ_ (LSCF), Ba_0.5_Sr_0.5_Co_0.8_Fe_0.2_O_3-δ_ (BSCF), Sm_0.5_Sr_0.5_CoO_3-δ_ (SSC), BaCo_0.4_Fe_0.4_Zr_0.1_Y_0.1_O_3-δ_ (BCFZY), and PrBa_0.5_Sr_0.5_Co_1.5_Fe_0.5_O_5+δ_ (PBSCF)

| **State-of-the-art catalysts** | **Cost ($/kg)** | ***R*_p_ @ 600 °C (Ω cm^2^)** | ***G*_hull­_ (eV/atom)** | **Ref** |
| --- | --- | --- | --- | --- |
| LSCF | 138.02 | 1.302, 1.406, 1.223 | 0.4946 | ^23, 38, 39^ |
| BSCF | 8.87 | 0.078,0.108, 0.029 | 0.3992 | ^2, 40, 41^ |
| PBC | 163.90 | 0.142, 0.081, 0.1062 | 0.1669 | ^42-44^ |
| SSC | 110.12 | 0.348,0.254, | 0.1833 | ^3, 45^ |
| BCFZY | 23.95 | 0.285 | 0.3476 | ^4^ |
| PBSCF | 170.98 | 0.108 | 0.2615 | ^46^ |

Table S11. Counterfactual analysis showing the predicted change in log(*R*_p_) upon ±1 standard deviation perturbation of *H_dp_* or DensificationIndex, with all other features held constant

| **Feature** | **Perturbation** | **Mean Δlog (*R*_p_)** |
| --- | --- | --- |
| *H_dp_* | +1 std | -0.067 |
| *H_dp_* | -1 std | +0.143 |
| DensificationIndex | +1 std | -0.024 |
| DensificationIndex | -1 std | +0.226 |

Table S12. Summary of novel promising candidates identified by the TSMS model, including their predicted *G*_hull_ and *R*_p_, validated through standardized experiments

| **No.** | **Candidate** | **Predicted *G*_hull_ (eV/atom)** | **Predicted *R*_p_ @ 600°C (Ω cm2)** | **Experimental *R*_p_ @ 600°C (Ω cm2)** |
| --- | --- | --- | --- | --- |
| 1 | BaCo_0.75_Hf_0.125_Zr_0.125_O_3_ | 0.1291 | 0.07341 | 0.070 |
| 2 | BaCo_0.5_Hf_0.25_Zr_0.25_O_3_ | 0.0859 | 0.08627 | 0.081 |
| 3 | BaCo_0.875_Mo_0.0625_Nb_0.0625_O_3_ | 0.1784 | 0.08160 | 0.080 |
| 4 | BaCo_0.75_Mo_0.125_Nb_0.125_O_3_ | 0.1220 | 0.09392 | 0.092 |
| 5 | BaCo_0.875_Sc_0.0625_Y_0.0625_O_3_ | 0.1912 | 0.07890 | 0.077 |
| 6 | BaCo_0.75_Sc_0.125_Y_0.125_O_3_ | 0.1814 | 0.06859 | 0.070 |
| 7 | BaCo_0.75_Hf_0.125_Dy_0.125_O_3_ | 0.1802 | 0.09396 | 0.092 |
| 8 | BaCo_0.75_Ta_0.1875_Y_0.0625_O_3_ | 0.1147 | 0.09368 | 0.092 |
| 9 | BaCo_0.625_Nb_0.1875_Ta_0.1875_O_3_ | 0.0908 | 0.09740 | 0.097 |
| 10 | BaCo_0.75_Ta_0.125_Sn_0.125_O_3_ | 0.1802 | 0.08762 | 0.078 |
| 11 | BaCo_0.875_Sc_0.0625_Hf_0.0625_O_3_ | 0.1912 | 0.09061 | 0.100 |
| 12 | BaCo_0.875_Nb_0.0625_Zr_0.0625_O_3_ | 0.1900 | 0.08201 | 0.079 |
| 13 | BaCo_0.75_Nb_0.125_Zr_0.125_O_3_ | 0.1291 | 0.07147 | 0.074 |
| 14 | BaCo_0.625_Sc_0.1875_Y_0.1875_O_3_ | 0.1003 | 0.07903 | 0.080 |
| 15 | BaCo_0.75_W_0.125_Dy_0.125_O_3_ | 0.1566 | 0.09104 | 0.096 |
| 16 | BaCo_0.875_Nb_0.0625_Sc_0.0625_O_3_ | 0.1901 | 0.07844 | 0.078 |
| 17 | BaCo_0.75_Nb_0.125_Sc_0.125_O_3_ | 0.1638 | 0.07379 | 0.076 |
| 18 | BaCo_0.875_Mo_0.0625_Y_0.0625_O_3_ | 0.1937 | 0.08867 | 0.086 |
| 19 | BaCo_0.75_Mo_0.125_Y_0.125_O_3_ | 0.1638 | 0.08502 | 0.084 |
| 20 | BaCo_0.75_Sb_0.125_Y_0.125_O_3_ | 0.1847 | 0.08067 | 0.084 |
| 21 | BaCo_0.75_Ho_0.125_W_0.125_O_3_ | 0.1517 | 0.08997 | 0.088 |
| 22 | BaCo_0.75_Dy_0.125_Nb_0.125_O_3_ | 0.1638 | 0.08677 | 0.088 |
| 23 | BaCo_0.75_Sc_0.125_Dy_0.125_O_3_ | 0.1649 | 0.06621 | 0.070 |
| 24 | BaCo_0.75_Sb_0.125_Zr_0.125_O_3_ | 0.1802 | 0.08532 | 0.087 |
| 25 | BaCo_0.75_Sb_0.125_Dy_0.125_O_3_ | 0.1847 | 0.09371 | 0.096 |

Table S13. Summary of Rietveld refinement results from high-temperature XRD under air and wet air conditions

| **Air** |  |  |  | **Wet Air** |  |  |  |
| --- | --- | --- | --- | --- | --- | --- | --- |
| **Temperature (°C)** | **R_wp_ (%)** | **R_p_ (%)** | ***a* (Å)** | **Temperature (°C)** | **R_wp_ (%)** | **R_p_ (%)** | ***a* (Å)** |
| 50 | 14.52 | 11 | 4.082 | 50 | 16.33 | 12.32 | 4.0901 |
| 100 | 15.28 | 11.56 | 4.0851 | 100 | 16.73 | 12.72 | 4.0905 |
| 150 | 15.86 | 11.86 | 4.0883 | 150 | 16.98 | 12.96 | 4.0919 |
| 200 | 15.87 | 12.06 | 4.0916 | 200 | 16.8 | 12.76 | 4.0942 |
| 250 | 15.23 | 11.64 | 4.0934 | 250 | 16.46 | 12.5 | 4.0953 |
| 300 | 15.81 | 12.07 | 4.0976 | 300 | 16.59 | 12.24 | 4.0986 |
| 350 | 15.69 | 11.75 | 4.10206 | 350 | 17.23 | 12.76 | 4.1028 |
| 400 | 16.25 | 12.31 | 4.10573 | 400 | 16.59 | 12.2 | 4.1066 |
| 450 | 16.21 | 12.2 | 4.11017 | 450 | 16.78 | 12.57 | 4.1105 |
| 500 | 16.54 | 12.47 | 4.11411 | 500 | 16.03 | 11.72 | 4.11447 |
| 550 | 16.22 | 11.91 | 4.1181 | 550 | 15.63 | 11.72 | 4.11826 |
| 600 | 17.22 | 12.79 | 4.1214 | 600 | 16.13 | 12.16 | 4.12196 |
| 650 | 16.58 | 12.13 | 4.12562 | 650 | 15.25 | 11.28 | 4.12555 |
| 700 | 15.38 | 11.34 | 4.1296 | 700 | 16.26 | 12.17 | 4.12954 |

Table S14. Summary of vibrational frequencies for ORR intermediates on the BO_2_-terminated (001) surface of BCNZ20. (Unit: cm^-1^)

| $\mathbf{(}\boldsymbol{O}_{\mathbf{Ⅰ}}^{\boldsymbol{q}_{\mathbf{Ⅰ}}}\boldsymbol{\ldots}\boldsymbol{O}_{\mathbf{Ⅱ}}^{\boldsymbol{q}_{\mathbf{Ⅱ}}}\mathbf{)*}$ | $\boldsymbol{(}\boldsymbol{O}_{\mathbf{Ⅰ}}^{\boldsymbol{q}_{\mathbf{Ⅰ}}^{\boldsymbol{'}}}\boldsymbol{)*}$ | $\left( \boldsymbol{O}_{\mathbf{Ⅰ}}^{\boldsymbol{q}_{\mathbf{Ⅰ}}^{\mathbf{'}}} \right)\boldsymbol{*\ldots}\boldsymbol{OH}_{\boldsymbol{O}\mathbf{,surf}}^{\boldsymbol{\cdot}}$ | $\left( \boldsymbol{OH}_{\mathbf{Ⅰ}}^{\boldsymbol{q}_{\mathbf{Ⅰ}}^{\mathbf{'''}}} \right)\mathbf{*}$ | $\left( \boldsymbol{OH}_{\mathbf{Ⅰ}}^{\boldsymbol{q}_{\mathbf{Ⅰ}}^{\mathbf{'''}}} \right)\boldsymbol{*\ldots}\boldsymbol{OH}_{\boldsymbol{O}\mathbf{,surf}}^{\boldsymbol{\cdot}}$ |
| --- | --- | --- | --- | --- |
| 1158.3 | 679.3 | 3572.2 | 3577.2 | 3645.0 |
| 405.3 | 404.5 | 892.7 | 929.9 | 3528.1 |
| 246.5 | 88.7 | 735.4 | 555.4 | 925.6 |
| 145.6 |  | 514.3 | 496.5 | 877.5 |
| 89.7 |  | 331.9 | 253.8 | 565.6 |
| 21.6 |  | 182.2 | 115.6 | 480.6 |
|  |  |  |  | 417.0 |
|  |  |  |  | 139.9 |
|  |  |  |  | 104.0 |

Table S15. Summary of reported high-performance P-SOCs and their electrochemical performance under fuel cell mode

| **Configuration** | **Atmosphere** | **Peak Power Density (W cm^2^)** | | | | | **Ref** |
| --- | --- | --- | --- | --- | --- | --- | --- |
|  |  | **600°C** | **550°C** | **500°C** | **450°C** | **400°C** |  |
| BCNZ20\|BSCYb172(5.8 μm)\|BSCYb172-NiO | Oxygen\|\|3%H_2_O-97%H_2_ | 2.68 | 2.24 | 1.35 | 0.80 | 0.41 | This work |
| BCNZ20\|BSCYb172(5.8 μm)\|BSCYb172-NiO | Air\|\|3%H_2_O-97%H_2_ | 2.00±0.06 | 1.63 | 1.00 | 0.62 | 0.35 | This work |
| PBCHf\|BSCYb172(7 μm)\|BSCYb172-NiO | Air\|\|3%H_2_O-97%H_2_ | 1.49 | 1.29 | 0.93 | 0.6 |  | ^14^ |
| BPHYC\|BSCYb172(10 μm)\|BSCYb172-NiO | Oxygen\|\|3%H_2_O-97%H_2_ |  |  | 1.05 |  |  | ^28^ |
| BPHYC\|BSCYb172(10 μm)\|BSCYb172-NiO | Air\|\|3%H_2_O-97%H_2_ | 1.57 | 1.21 | 0.82 | 0.52 |  | ^28^ |
| BCZY63+BCFZY\|BZCYYb1711(26.4 μm)\|BZCYYb-NiO | Air\|\|H_2_ | 0.64 | 0.53 | 0.45 |  |  | ^4^ |
| PBSCF\|PBSCF(PLD film)\|BZCYYb4411(12.2 μm)\|BZCYYb4411-NiO | Air\|\|3%H_2_O-97%H_2_ | 1.085 | 0.822 | 0.593 | 0.372 |  | ^47^ |
| BSC+PBSCF\|BZCYYb1711(3 μm)\|BZCYYb1711-NiO | Oxygen\|\|3%H_2_O-97%H_2_ |  | 1.47 | 0.98 | 0.78 |  | ^48^ |
| BSC+PBSCF\|BZCYYb1711(3 μm)\|BZCYYb1711-NiO | Air\|\|3%H_2_O-97%H_2_ |  | 1.22 | 0.83 | 0.55 |  | ^48^ |
| BFZ\|BZCYYb1711(3.7 μm)\|BZCYYb1711-NiO | Air\|\|3%H_2_O-97%H_2_ | 1.28 | 0.95 | 0.67 |  |  | ^49^ |
| PNC\|BZCYYb1711(16 μm)\|BZCYYb1711-NiO | Oxygen\|\|3%H_2_O-97%H_2_ | 1.6 | 1.25 | 0.90 |  |  | ^50^ |

Table S16. Summary of open circuit voltages (OCVs), ohmic resistances (*R*_o_), and polarization resistances (*R*_p_) measured under air and oxygen atmospheres at temperatures from 600 to 400 °C

|  | **Atmosphere** | | | | | |
| --- | --- | --- | --- | --- | --- | --- |
|  | **Air\|\|3%H_2_O-97%H_2_** | | | **Oxygen\|\|3%H_2_O-97%H_2_** | | |
| **Temperature**  **(°C)** | **OCV (V)** | ***R*_o_ (Ω cm^2^)** | ***R*_p_ (Ω cm^2^)** | **OCV (V)** | ***R*_o_ (Ω cm^2^)** | ***R*_p_ (Ω cm^2^)** |
| 600 | 1.08 | 0.075 | 0.072 | 1.09 | 0.069 | 0.050 |
| 550 | 1.11 | 0.087 | 0.165 | 1.13 | 0.083 | 0.121 |
| 500 | 1.12 | 0.116 | 0.579 | 1.14 | 0.105 | 0.373 |
| 450 | 1.15 | 0.177 | 1.366 | 1.17 | 0.176 | 0.972 |
| 400 | 1.16 | 0.299 | - | 1.18 | 0.276 | - |

Movie S1.

Visualization of proton transport pathways from AIMD in a 4*a*×4*a*×4*a* BCNZ20 supercell (64 Ba, 52 Co, 6 Nb, 6 Zr, 192 O, 2 H) over 250 ps.

Data S1. (dataset_1_20250319.csv)

Training dataset for the Stage Ⅰ machine learning model.

Data S2. (dataset_2_20250319.csv)

Training dataset for the Stage Ⅱ active learning model.

References

1.	S. J. Skinner, *Int J Inorg Mater*, 2001, **3**, 113-121.

2.	Z. P. Shao and S. M. Haile, *Nature*, 2004, **431**, 170-173.

3.	C. R. Xia, W. Rauch, F. L. Chen and M. L. Liu, *Solid State Ionics*, 2002, **149**, 11-19.

4.	C. C. Duan, J. H. Tong, M. Shang, S. Nikodemski, M. Sanders, S. Ricote, A. Almansoori and R. O'Hayre, *Science*, 2015, **349**, 1321-1326.

5.	S. Choi, T. C. Davenport and S. M. Haile, *Energ Environ Sci*, 2019, **12**, 206-215.

6.	C. J. Bartel, C. Sutton, B. R. Goldsmith, R. H. Ouyang, C. B. Musgrave, L. M. Ghiringhelli and M. Scheffler, *Sci Adv*, 2019, **5**.

7.	Z. H. Li, X. Mao, D. S. Feng, M. R. Li, X. Y. Xu, Y. D. Luo, L. Z. Zhuang, R. J. Lin, T. J. Zhu, F. L. Liang, Z. Huang, D. Liu, Z. F. Yan, A. J. Du, Z. P. Shao and Z. H. Zhu, *Nat Commun*, 2024, **15**.

8.	P. E. Blochl, *Phys Rev B*, 1994, **50**, 17953-17979.

9.	G. Kresse and J. Furthmuller, *Comp Mater Sci*, 1996, **6**, 15-50.

10.	J. P. Perdew, K. Burke and M. Ernzerhof, *Phys Rev Lett*, 1996, **77**, 3865-3868.

11.	A. Jain, S. P. Ong, G. Hautier, W. Chen, W. D. Richards, S. Dacek, S. Cholia, D. Gunter, D. Skinner, G. Ceder and K. A. Persson, *Apl Mater*, 2013, **1**.

12.	C. Chen and S. P. Ong, *Nat Comput Sci*, 2022, **2**, 718-+.

13.	V. Somjit and B. Yildiz, *Acs Appl Mater Inter*, 2022, **14**, 42613-42627.

14.	X. Y. Hu, Y. C. Zhou, Z. Y. Luo, H. Y. Li, N. Shi, Z. J. Liu, W. L. Zhang, W. N. Wang, Y. Ding and M. L. Liu, *Energ Environ Sci*, 2024, DOI: 10.1039/d4ee03762f.

15.	M. Aykol, S. Kim, V. I. Hegde, D. Snydacker, Z. Lu, S. Q. Hao, S. Kirklin, D. Morgan and C. Wolverton, *Nat Commun*, 2016, **7**.

16.	Y. P. Cao, M. J. Gadre, A. T. Ngo, S. B. Adler and D. D. Morgan, *Nat Commun*, 2019, **10**.

17.	R. Nelson, C. Ertural, J. George, V. L. Deringer, G. Hautier and R. Dronskowski, *J Comput Chem*, 2020, **41**, 1931-1940.

18.	X. Hu, Y. Xie, Y. Wan, Y. Yang, X. Wu and C. Xia, *Applied Catalysis B: Environmental*, 2021, **286**, 119901.

19.	Y. Zhou, E. Liu, Y. Chen, Y. Liu, L. Zhang, W. Zhang, Z. Luo, N. Kane, B. Zhao and L. Soule, *Acs Energy Lett*, 2021, **6**, 1511-1520.

20.	Z. Liu, D. Cheng, Y. Zhu, M. Liang, M. Yang, G. Yang, R. Ran, W. Wang, W. Zhou and Z. Shao, *Chem Eng J*, 2022, **450**, 137787.

21.	K. Pei, S. Luo, F. He, J. Arbiol, Y. Xu, F. Zhu, Y. Wang and Y. Chen, *Applied Catalysis B: Environmental*, 2023, **330**, 122601.

22.	C. Duan, R. Kee, H. Zhu, N. Sullivan, L. Zhu, L. Bian, D. Jennings and R. O’Hayre, *Nat Energy*, 2019, **4**, 230-240.

23.	Y. Niu, Y. Zhou, W. Zhang, Y. Zhang, C. Evans, Z. Luo, N. Kane, Y. Ding, Y. Chen and X. Guo, *Adv Energy Mater*, 2022, **12**, 2103783.

24.	K. Pei, Y. Zhou, K. Xu, H. Zhang, Y. Ding, B. Zhao, W. Yuan, K. Sasaki, Y. Choi and Y. Chen, *Nat Commun*, 2022, **13**, 2207.

25.	F. He, Y. Zhou, T. Hu, Y. Xu, M. Hou, F. Zhu, D. Liu, H. Zhang, K. Xu and M. Liu, *Adv Mater*, 2023, **35**, 2209469.

26.	F. He, S. Liu, T. Wu, M. Yang, W. Li, G. Yang, F. Zhu, H. Zhang, K. Pei and Y. Chen, *Adv Funct Mater*, 2022, **32**, 2206756.

27.	M. Saqib, I.-G. Choi, H. Bae, K. Park, J.-S. Shin, Y.-D. Kim, J.-I. Lee, M. Jo, Y.-C. Kim and K.-S. Lee, *Energ Environ Sci*, 2021, **14**, 2472-2484.

28.	Z. Luo, X. Hu, Y. Zhou, Y. Ding, W. Zhang, T. Li and M. Liu, *Adv Mater*, 2024, 2311159.

29.	Y. C. Zhou, E. Z. Liu, Y. Chen, Y. C. Liu, L. Zhang, W. L. Zhang, Z. Y. Luo, N. Kane, B. Zhao, L. Soule, Y. H. Niu, Y. Ding, H. P. Ding, D. Ding and M. L. Liu, *Acs Energy Lett*, 2021, **6**, 1511-1520.

30.	W. K. Ye, C. Chen, Z. B. Wang, I. H. Chu and S. P. Ong, *Nat Commun*, 2018, **9**.

31.	J. Schmidt, J. M. Shi, P. Borlido, L. M. Chen, S. Botti and M. A. L. Marques, *Chem Mater*, 2017, **29**, 5090-5103.

32.	W. Li, R. Jacobs and D. Morgan, *Comp Mater Sci*, 2018, **150**, 454-463.

33.	C. Chen, W. K. Ye, Y. X. Zuo, C. Zheng and S. P. Ong, *Chem Mater*, 2019, **31**, 3564-3572.

34.	G. J. Cheng, X. G. Gong and W. J. Yin, *Nat Commun*, 2022, **13**.

35.	J. Schmidt, L. Pettersson, C. Verdozzi, S. Botti and M. A. L. Marques, *Sci Adv*, 2021, **7**.

36.	S. Zhai, H. P. Xie, P. Cui, D. Q. Guan, J. Wang, S. Y. Zhao, B. Chen, Y. F. Song, Z. P. Shao and M. Ni, *Nat Energy*, 2022, **7**, 866-875.

37.	R. Jacobs, J. Liu, H. Abernathy and D. Morgan, *Adv Energy Mater*, 2024, **14**.

38.	S. A. M. Ali, M. Anwar, N. F. Raduwan, A. Muchtar and M. R. Somalu, *J Sol-Gel Sci Techn*, 2018, **86**, 493-504.

39.	M. F. Liu, D. Ding, K. Blinn, X. X. Li, L. F. Nie and M. Liu, *Int J Hydrogen Energ*, 2012, **37**, 8613-8620.

40.	M. R. Li, M. W. Zhao, F. Li, W. Zhou, V. K. Peterson, X. Y. Xu, Z. P. Shao, I. Gentle and Z. H. Zhu, *Nat Commun*, 2017, **8**.

41.	Q. L. Liu, K. A. Khor and S. H. Chan, *J Power Sources*, 2006, **161**, 123-128.

42.	Y. H. Wan, Y. L. Xing, Y. H. Li, D. M. Huan and C. R. Xia, *J Power Sources*, 2018, **402**, 363-372.

43.	Y. Wang, H. Zhang, F. L. Chen and C. R. Xia, *J Power Sources*, 2012, **203**, 34-41.

44.	H. X. Gu, G. M. Yang, Y. Hu, M. Z. Liang, S. H. Chen, R. Ran, M. G. Xu, W. Wang, W. Zhou and Z. P. Shao, *Int J Hydrogen Energ*, 2020, **45**, 25996-26004.

45.	H. Zhang, F. Zhao, F. L. Chen and C. R. Xia, *Solid State Ionics*, 2011, **192**, 591-594.

46.	S. Park, S. Choi, J. Shin and G. Kim, *Rsc Adv*, 2014, **4**, 1775-1781.

47.	S. Choi, C. J. Kucharczyk, Y. G. Liang, X. H. Zhang, I. Takeuchi, H. I. Ji and S. M. Haile, *Nat Energy*, 2018, **3**, 202-210.

48.	F. Liu, H. Deng, D. Diercks, P. Kumar, M. H. A. Jabbar, C. Gumeci, Y. Furuya, N. Dale, T. Oku, M. Usuda, P. Kazempoor, L. Y. Fang, D. Chen, B. Liu and C. C. Duan, *Nat Energy*, 2023, DOI: 10.1038/s41560-023-01350-4.

49.	Z. Wang, Y. H. Wang, J. Wang, Y. F. Song, M. J. Robson, A. Seong, M. T. Yang, Z. Q. Zhang, A. Belotti, J. P. Liu, G. Kim, J. Lim, Z. P. Shao and F. Ciucci, *Nat Catal*, 2022, **5**, 777-787.

50.	W. J. Bian, W. Wu, B. M. Wang, W. Tang, M. Zhou, C. R. Jin, H. P. Ding, W. W. Fan, Y. H. Dong, J. Li and D. Ding, *Nature*, 2022, **604**, 479-+.
